# Sex-dependent effects of genetic upregulation of activated protein C on delayed effects of acute radiation exposure in the mouse heart, small intestine, and skin

**DOI:** 10.1371/journal.pone.0252142

**Published:** 2021-05-24

**Authors:** Vijayalakshmi Sridharan, Kristin A. Johnson, Reid D. Landes, Maohua Cao, Preeti Singh, Gail Wagoner, Abdallah Hayar, Emily D. Sprick, Kayla A. Eveld, Anusha Bhattacharyya, Kimberly J. Krager, Nukhet Aykin-Burns, Hartmut Weiler, Jose A. Fernández, John H. Griffin, Marjan Boerma

**Affiliations:** 1 Department of Pharmaceutical Sciences, Division of Radiation Health, University of Arkansas for Medical Sciences, Little Rock, AR, United States of America; 2 College of Pharmacy, University of Arkansas for Medical Sciences, Little Rock, AR, United States of America; 3 Department of Biostatistics, University of Arkansas for Medical Sciences, Little Rock, AR, United States of America; 4 College of Dentistry, Texas A&M University, Dallas, TX, United States of America; 5 Department of Neurobiology & Developmental Sciences, University of Arkansas for Medical Sciences, Little Rock, AR, United States of America; 6 Versiti and the Medical College of Wisconsin, Milwaukee, WI, United States of America; 7 Department of Molecular Medicine, Scripps Research, La Jolla, CA, United States of America; Northwestern University Feinberg School of Medicine, UNITED STATES

## Abstract

Accidental exposure to ionizing radiation may lead to delayed effects of acute radiation exposure (DEARE) in many organ systems. Activated protein C (APC) is a known mitigator of the acute radiation syndrome. To examine the role of APC in DEARE, we used a transgenic mouse model with 2- to 3-fold increased plasma levels of APC (high in APC, APCHi). Male and female APCHi mice and wild-type littermates were exposed to 9.5 Gy γ-rays with their hind-legs (bone marrow) shielded from radiation to allow long-term survival. At 3 and 6 months after irradiation, cardiac function was measured with ultrasonography. At 3 months, radiation increased cardiac dimensions in APCHi males, while decreases were seen in wild-type females. At this early time point, APCHi mice of both sexes were more susceptible to radiation-induced changes in systolic function compared to wild-types. At 6 months, a decrease in systolic function was mainly seen in male mice of both genotypes. At 6 months, specimens of heart, small intestine and dorsal skin were collected for tissue analysis. Female APCHi mice showed the most severe radiation-induced deposition of cardiac collagens but were protected against a radiation-induced loss of microvascular density. Both male and female APCHi mice were protected against a radiation induced upregulation of toll-like receptor 4 in the heart, but this did not translate into a clear protection against immune cell infiltration. In the small intestine, the APCHi genotype had no effect on an increase in the number of myeloperoxidase positive cells (seen mostly in females) or an increase in the expression of T-cell marker CD2 (males). Lastly, both male and female APCHi mice were protected against radiation-induced epidermal thickening and increase in 3-nitrotyrosine positive keratinocytes. In conclusion, prolonged high levels of APC in a transgenic mouse model had little effects on indicators of DEARE in the heart, small intestine and skin, with some differential effects in male compared to female mice.

## Introduction

Military personnel, nuclear facility workers and the general population continue to be at risk for exposure to ionizing radiation due to a radiological event or incident. Total or partial body exposures to ionizing radiation can lead to the acute radiation syndrome (ARS), the outcome of which is mainly determined by bone marrow failure and injuries in the gastrointestinal tract. Individuals who survive the ARS can experience delayed effects of acute radiation exposure (DEARE) that lead to chronic dysfunction in organ systems within months to years after irradiation. Many organs are known to undergo long-term adverse remodeling, including the heart, small intestine and skin [1–6].

As radiological events are unpredictable and may involve a large number of victims, radiation mitigators are needed to reduce both acute and delayed radiation toxicity when administered 24 hours after exposure. Currently, only a small number of such radiation mitigators have been approved by the FDA: human recombinant granulocyte colony–stimulating factors (G-CSF), granulocyte-macrophage colony stimulating factor (GM-CSF), sargramostim (Leukine), and the thrombopoietin analog romiplastin (Nplate) [7–9]. All countermeasures approved thus far are aimed at enhancing the function of acutely-suppressed bone marrow and therefore target mostly the hematopoietic acute radiation syndrome (H-ARS). There are no FDA approved countermeasures against gastrointestinal radiation injury or DEARE in various organ systems. Hence, research is needed to provide insight into the mechanisms by which ionizing radiation causes prolonged radiation injuries. Once identified, such mechanisms could reveal additional pathways to be targeted by radiation mitigators.

Endothelial and vascular injury are thought to play a central role in radiation-induced tissue damage. In the acute situation, radiation causes reduced endothelial barrier function, endothelial cell apoptosis, expression of chemokines and adhesion molecules, and loss of vascular thromboresistance [10, 11]. Vascular dysfunction, which becomes apparent months to years after initial radiation exposure includes a decrease in microvascular density, perivascular fibrosis, and vascular wall thickening [12–14]. A loss of thrombomodulin is a prominent feature of post-radiation endothelial dysfunction [10]. Normally, thrombomodulin is a transmembrane glycoprotein located on the luminal surface of endothelial cells in most blood vessels. Thrombin, when bound to thrombomodulin, loses its procoagulant, pro-inflammatory, and profibrogenic effects and, instead, acquires the ability to activate protein C. Activated protein C (APC) limits further thrombin generation and counteracts thrombin’s adverse effects [15]. In addition, APC is a critical component in plasma that has potent intrinsic anti-coagulant, anti-inflammatory and cytoprotective properties [16] and it modifies endothelial function via several cell surface receptors [17, 18]. A radiation-induced loss of thrombomodulin is thought to generate a prothrombotic and profibrogenic environment, in part through reducing the production of APC [10]. Along these lines, we have previously shown that recombinant APC is an effective mitigator of acute radiation injury and death when administered beginning at 24 hours after total body irradiation in a mouse model [19]. However, the role of APC in the development of DEARE is unknown.

We previously generated a mouse model on a C57BL/6N background that expresses the D168F/N173K mouse analogue of the hyperactivatable human D167F/D172K protein C variant [20], resulting in a 2- to 3-fold increase in plasma levels of murine APC (mice with high APC, APCHi) [21]. This hyperactivatable form of protein C shows the same activity as wild-type APCs [20]. In this study, we use male and female APCHi mice and wild-type littermates to examine the role of APC in delayed radiation injury in the heart, small intestine and epidermis.

## Materials and methods

### APCHi animal model

All procedures were approved by the Institutional Animal Care and Use Committee of the University of Arkansas for Medical Sciences (UAMS) under protocol #3763. Male and female mice carrying the D168F/N173K transgene (obtained from Hartmut Weiler, PhD, BloodCenter of Wisconsin) were bred with wild-type C57BL/6N mice (Jackson Laboratories, Bar Harbor, ME) to obtain APCHi and wild-type mice. DNA was isolated from a tail snip, and primers (5’ CAAGCCGGTTTACTCTGACCC 3’ and 5’ CCTTGGAAATGGTTCCAGTTCATCTTCTA 3’) were used to detect the transgene [21]. All mice were maintained in the UAMS Division of Laboratory Animal Medicine on a 12:12 light-to-dark cycle with free access to standard rodent chow and water.

### Animal total body irradiation and 30-day survival

At the age of 12–14 weeks, APCHi and wild-type mice of both sexes (35 mice in total) were exposed 9.5 Gy dose of γ-rays delivered at 1 Gy/min–the dose used in our prior studies of APC administration in acute radiation injury [19], using a ^137^Cs source cabinet irradiator (Mark 1, Model 68A, JL Shepherd & Associates, San Fernando, CA) without the use of anesthesia. Dosimetry was performed with Gafchromic film (DOSE-MAP, Ashland Specialty Ingredients, Wayne, NJ) and an ion chamber (Exradin A20, Standard Imaging, Middleton, WI) and electrometer (X4000, Standard Imaging) that are calibrated for γ-rays once a year. Animals were returned to their home cages and observed at least once a day for 30 days to monitor survival.

### Animal irradiation with hind-leg shielding and long-term follow-up

At the age of 12–14 weeks, APCHi and wild-type mice of both sexes (149 mice in total) were randomized to receive either sham irradiation or a 9.5 Gy dose of γ-rays delivered at 1 Gy/min. We developed an irradiation model in which both hind-legs of the mice were shielded from radiation, to allow for bone marrow recovery and long-term post-irradiation survival of the animals. For this purpose, unanesthetized mice were placed in Plexiglas mouse holders on a Plexiglas base plate, and blocks of 2 cm thick tungsten (4 half-value layers) were carefully placed over both hind-legs of the animals. The animals were then placed in a ^137^Cs source cabinet irradiator (Mark 1, Model 68A, JL Shepherd & Associates) for 9.5 minutes. Additional mice were exposed to sham-irradiation by bringing them to the radiation room and placing them in the holders for 9.5 minutes without exposing them to radiation. Dosimetry was performed as described above.

Animals were monitored daily by trained animal care technicians and laboratory staff for signs of radiation sickness or distress as instructed by institutional veterinarians. Criteria to determine when animals should be euthanized were hunched position, lethargy, and significant weight loss. None of the animals reached these humane endpoints. All 149 animals survived until 6 months after irradiation.

### Cardiac function

At 3 and 6 months after irradiation, *in vivo* cardiac function was assessed with ultrasonography. Mice were examined in random order, and the sonographer was blinded to experimental group. Because anesthesia is known to alter cardiac physiology [22], we developed a procedure in which mice were under anesthesia for the shortest possible time during ultrasonography [23]. Hence, the day before ultrasonography, mice were anesthetized with 2% isoflurane, and hair was removed from the thorax and abdomen with depilatory cream. On the day of ultrasonography, mice were anesthetized with 1.5% isoflurane and placed supine on a heated platform that monitors respiration rate and generates an electrocardiogram. The mice were scanned immediately with a Vevo® 2100 high-resolution pre-clinical small animal imaging system (VisualSonics, Toronto, ON) with a MS400 (18–38 MHz) transducer. Echocardiograms were obtained in the short axis M-mode at the mid-left ventricular level. Pulsed-wave Doppler was used to determine mitral valve E and A velocities in a four-chamber view of the heart. The Vevo® 2100 software version 1.6.0 cardiac package was used to obtain echocardiographic parameters from three M-mode scans per animal. The vascular package was used to assess mitral valve E and A velocities and blood flow parameters from 3 consecutive wave patterns per pulsed-wave Doppler scan, in up to 3 scans per animal. The measurements from multiple scans per animal were averaged to obtain one value per parameter per animal.

### Heart rate

To assess potential effects of radiation on heart rhythm, at 6 months after irradiation in female mice, electrocardiograms (ECG) were obtained from unanesthetized mice. For this purpose, mice were briefly restrained in a cone-shaped transparent bag (Braintree Scientific Inc., Braintree, MA). A force transducer in the form of a ~8 mm diameter rod (Fort100, connected to a BRIDGE8 amplifier unit, World Precision Instruments, Sarasota, FL) was placed in between the bag and the abdomen of the mouse to monitor respiration. Two 25G needles were inserted under the skin on both sides of the abdomen. A tungsten wire (~250 μm diameter), serving as ECG probe, was inserted through each needle into the skin, and then the needles were withdrawn. The ECG probes were connected to an ISO-DAM8A DC amplifier, and both ECG and respiration probes were connected to a bioamplifier system (ISDB-8, World Precision Instruments). The settings on the ISO-DAM8A module were these: high-pass filter 3 kHz, low pass filter 0.1 Hz, DC offset off and gain X1000. The settings for recording of respiration were low-pass filter 7 kHz, offset high and gain X500. Both respiratory and ECG signals were acquired using an analog-to-digital converter (Digidata 1440A, Molecular Devices, Sunnyvale, CA). Signals were digitized at 5 kHz using pClamp software (Molecular Devices) and the detection of heart beat and breathing events was performed off-line with the Mini Analysis Program (Synaptosoft Inc. Decatur, GA). The parameters characterizing the events were then imported into OriginLab software (Microcal Software Inc., Northampton, MA) for analysis of heart rate.

### Tissue collection

At 6 months after irradiation, animals were anesthetized with 3% isoflurane inhalation, administered 30–40 U/kg heparin, and tissues were collected and immediately processed. The hearts were cut longitudinally, and one half of the heart was fixed in methanol Carnoy’s solution (60% methanol, 30% chloroform, 10% acetic acid). The remainder of the heart was dissected into atria, left and right ventricle and snap-frozen before storage at -80°C. The jejunum was cut longitudinally and fixed in methanol Carnoy’s solution. Separate specimens of jejunum were snap-frozen before storage at -80°C. Clippers were used to remove the hair from an area of the back, and specimens of skin were fixed in methanol Carnoy’s solution. Tissues were maintained in methanol Carnoy’s for 24 hours and then embedded in paraffin.

### Histology

All analyses were performed blinded to the experimental groups. For determination of collagen deposition, sections of heart, intestine and skin were deparaffinized, rehydrated, and incubated in Sirius Red supplemented with Fast Green. Sections were scanned with a ScanScope CS2 slide scanner and analyzed with ImageScope 12 software (Aperio, Leica Biosystems, Buffalo Grove, IL) or ImageJ software to determine the percentage of tissue area positive for collagens.

To visualize mast cells, deparaffinized and rehydrated sections of heart and intestine were incubated in 0.5% Toluidine Blue in 0.5 N HCl for 72 hours, followed by 0.7 N HCl for 10 minutes. Mast cells were counted using an Axioskop transmitted light microscope (Zeiss International, Oberkochen, Germany) and divided by total tissue area.

Cardiac microvascular density was determined from a lectin staining. Sections were deparaffinated and rehydrated, and then incubated in 1% H_2_O_2_ in methanol for 30 minutes to quench the endogenous peroxidase activity. Subsequently, sections were incubated with biotinylated Lycopersicon Esculentum (tomato) lectin (1:200, Vector Laboratories, Burlingame CA) for 90 minutes at room temperature. Lectin staining was visualized by incubating the sections in avidin-biotin-peroxidase complex (Vector Laboratories) for 45 minutes at room temperature and then in 0.5 mg/ml 3,3’-diaminobenzidine (DAB, Sigma-Aldrich, St. Louis, MO) in 0.3% H_2_O_2_ for 5 minutes. After counter staining with hematoxylin, sections were dehydrated and mounted. Sections were scanned with a ScanScope CS2 slide scanner and analyzed with ImageScope 12 software (Aperio). Four to five fields were randomly selected from each section to count the lectin stained capillaries, and the total numbers of capillaries were divided by the area of the field to obtain microvascular density.

The epidermal thickness of skin specimens was assessed with ImageJ software on Hematoxylin & Eosin stained sections. Five fields were randomly selected from each section and at least 10 measurements were taken perpendicular to the basement membrane in each field. The average value of 50 measurements of each section was used as single data point for analysis.

### Immunohistochemistry

Immunohistochemistry was used to assess expression of CD45 in the heart and intestine, α-smooth muscle cell (SMC) actin and von Willebrand factor (vWf) in the heart, myeloperoxidase (MPO) in intestine, and 4-hydroxynonenal (4-HNE) and 3-nitrotyrosine (3NT) in the epidermis. For this purpose, sections were rehydrated and incubated in 1% H_2_O_2_ in methanol to block endogenous peroxidase, followed by 10% normal serum added to 3% dry powdered milk and 0.2% bovine serum albumin in TBS to block non-specific antibody binding. Sections were incubated overnight at 4°C with antibodies and their concentrations as listed in **S1 Table**. After incubation with the primary antibodies, sections were incubated with biotinylated goat anti-rat IgG (1:400, Bio-Rad, Hercules, CA) or goat anti-rabbit IgG (1:400, Bio-Rad) for 30 min at room temperature, followed by an avidin-biotin-peroxidase complex (Vector Laboratories) for 45 minutes. Bound antibodies were visualized with 0.5 mg/mL DAB (Sigma-Aldrich) and counterstained with hematoxylin.

All stained sections were examined with an Axioskop transmitted-light microscope (Zeiss International), with the observer blinded to the experimental groups. CD45 positive cells were counted in 10 optical areas per section and divided by the total area of these 10 optical views. In a normal heart, α-SMC actin is only seen in SMC. In cardiac radiation fibrosis, spindle-shaped α-SMC actin positive cells appear (myofibroblasts) [24]. Normally, vWf is expressed on endothelial cells in mid-size and large arteries in the heart. In animal models of local heart irradiation, microvascular endothelial cells express vWf, which we see as an indication of endothelial dysfunction [14]. Heart sections stained for α-SMC actin and vWf were scored as shown in Table 1. In sections of intestine, MPO positive cells were counted and divided by total tissue area of each section. In sections of skin, 3NT positive cells were counted and divided by total epidermal tissue area. The area of the epidermis positive for 4-HNE staining was determined with ImageJ software.

**Table 1:** Criteria for numerical scoring of α-SMC actin and vWf immunohistochemistry.

	α-SMC actin	vWf
Score 0	Staining only in vascular SMC	Staining only on endothelial cells in larger arteries
Score 1	Between 1 and 10 positively stained myofibroblasts	vWf positive capillaries in one or two subsections of the heart
Score 2	Between 10 and 20 positively stained myofibroblasts	vWf positive capillaries in more than two subsections of the heart
Score 3	Between 20 and 100 positively stained myofibroblasts	vWf positive capillaries throughout the myocardium
Score 4	More than 100 positively stained myofibroblasts	Not applicable

### Immunoblotting

Immunoblot analysis was performed on frozen tissue samples of left ventricle or jejunum from 5–6 randomly selected males and 5–6 females for each genotype and radiation combination. A Potter-Elvehjem mechanical compact stirrer (BDC2002, Caframo LabSolutions, Georgian Bluffs, ON) was used to homogenize the tissue in a 1% Triton-X100 radio immunoprecipitation assay buffer containing protease inhibitors (1:100) and phosphatase inhibitors (1:100; Sigma-Aldrich). Protein concentration was determined with a bicinchoninic acid protein assay (Bio-Rad), and 25 μg protein was added to a 2x Laemmli buffer containing β-mercaptoethanol (5%). Gel electrophoresis was performed, and proteins were transferred to a polyvinylidene difluoride membrane. Membranes were incubated in primary antibodies at concentrations listed in **S1 Table** in TBS containing 0.1% Tween-20 and 5% non-fat dry milk at 4°C overnight. After incubating with horseradish peroxidase-conjugated secondary antibodies: goat anti-rabbit IgG (Cell Signaling Technology, Danvers, MA; 1:10,000), goat anti-rat IgG (Santa Cruz Biotechnology, Santa Cruz, CA; 1:30,000 or 1:40,000) or goat anti-mouse IgG (Jackson Immunoresearch, West Grove, PA; 1:20,000), membranes were covered in enhanced chemiluminescence Plus Western Blotting Detection Reagent (GE Healthcare Life Sciences, Chicago, IL) and placed on CL-Xposure Film (Thermo Scientific, Waltham, MA). Films were developed and imaged with an AlphaImager® gel documentation system (ProteinSimple, San Jose, CA). Densitometry was performed with ImageJ software. All proteins were normalized to the loading control glyceraldehyde 3-phosphate dehydrogenase (GAPDH).

### Statistical analyses

For outcomes collected only once on an animal (e.g., histological outcomes), we used analysis of variance (ANOVA) accounting for radiation, genotype, sex, and all 2- and 3-way interactions. Using residuals from the initial ANOVA, we evaluated normal assumptions with visual inspection of Q-Q plots and a battery of tests for normality, and evaluated constant variance assumptions with Levene’s test for homogeneous variances. Residuals from all outcomes were at least approximately normal in distribution. However, for several outcomes, we found variances to differ among experimental groups. Based on where differences were found among the experimental groups, we compared candidate covariance structures (e.g., all variances constant vs variances dependent upon radiation dose) with values of the Bayesian Information Criterion and likelihood ratio tests based on the restricted maximum likelihood (REML). For the chosen covariance structure, and thus final model, we estimated error degrees of freedom with Kenward-Roger’s method [25]. Our primary comparison of interest was the radiation×genotype interaction within a sex. This interaction compares the radiation effects in APCHi mice to the radiation effects in wild-type mice, hypothesizing radiation effects will be mitigated in the APCHi animals.

Ultrasonography outcomes were repeatedly measured on animals at 3 and 6 months. Further, heart rate [22] and body weight [26] can affect ultrasonography outcomes. While we did not see major changes in body weight in the study, we did include body weight as a covariate. Hence, to the ANOVA described above, we added time–a within-individual factor–and all subsequent 2-, 3-, and 4-way interactions, as well as the two covariates, heart rate and body weight; thus giving us a repeated measures analysis of covariance (ANCOVA). We chose the covariance structure accounting for repeated measures based on the Bayesian Information Criterion and REML likelihood ratio tests. As described above for the ANOVA, our primary focus was the radiation×genotype interaction within each sex at each month. The ANOVA and ANCOVA models were conducted in the MIXED procedure of SAS/STAT® software, version 9.4 (SAS Institute, Cary, NC). Because of the large number of outcomes and comparisons, we estimate the positive False Discovery Rate according to Storey’s 3^rd^ algorithm [27]. Statistical power considerations are presented in **S2 Table**. Statistical code used for the analyses is available upon request.

## Results

### Thirty-day radiation survival

There were no differences between male and female wild-type mice and APCHi mice in 30-day survival after total body exposure to 9.5 Gy γ-rays (**S1 Fig**).

### Animal characteristics in DEARE studies

All animals exposed to 9.5 Gy γ-rays with hind-leg shielding survived up to 6 months after irradiation. At 3 months after irradiation, wild-type animals showed a small loss of body weight compared to wild-type 0 Gy controls. At 6 months, all irradiated groups had a lower body weight than 0 Gy controls. In males, the effect of radiation was significantly more severe in wild-type compared to APCHi mice (**S2 Fig**).

### Effects of radiation in the heart

#### *In vivo* cardiac dimensions and function

**Fig 1:**
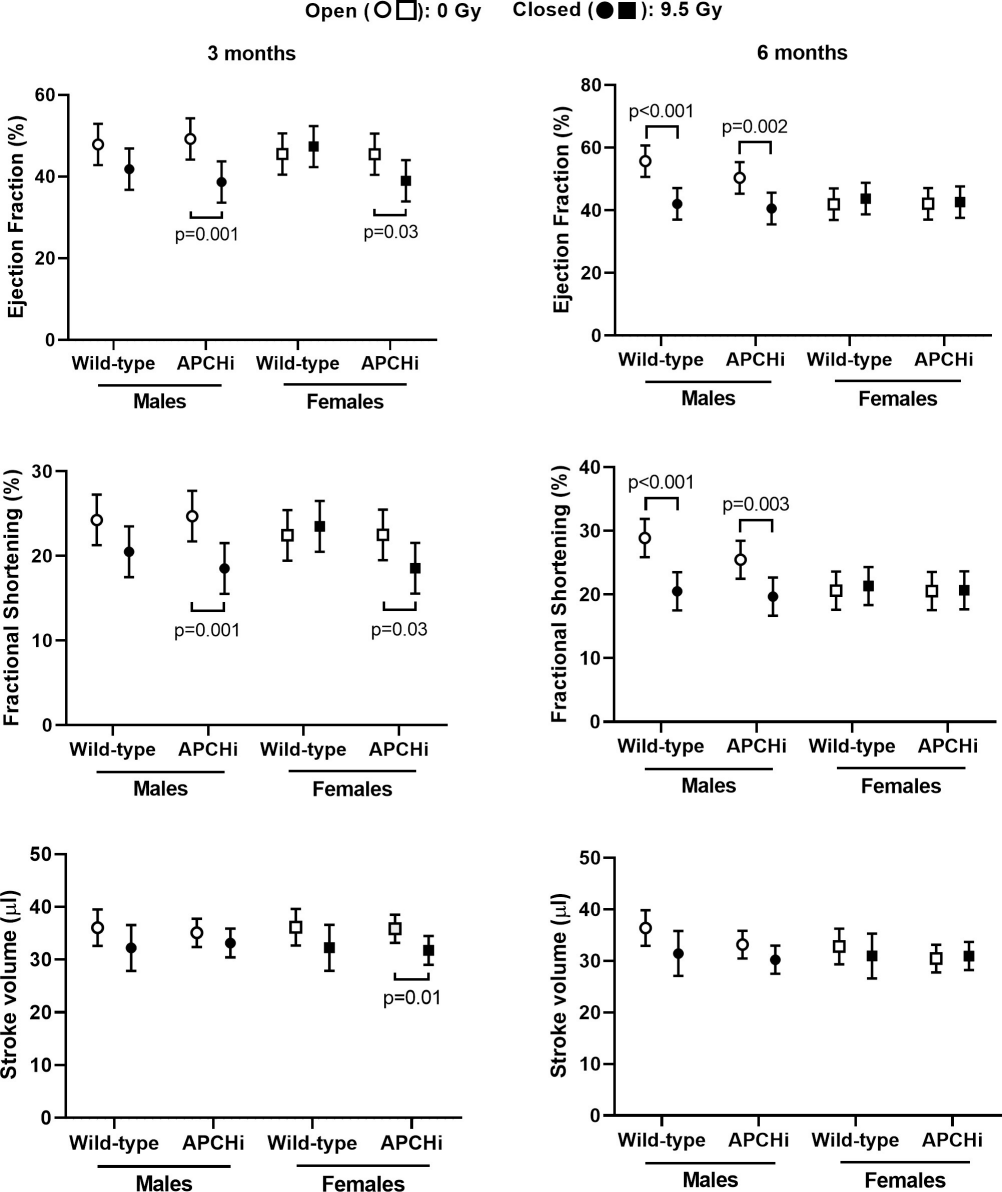
Left ventricular ejection fraction, fractional shortening and stroke volume at 3 and 6 months after irradiation. Means and standard deviations (SD) of the statistical model are shown; *n* = 9 wild-type males in 0 Gy, 12 wild-type males in 9.5 Gy, 8 APCHi males in 0 Gy, 10 APCHi males in 9.5 Gy, 8 wild-type females in 0 Gy, 8 wild-type females in 9.5 Gy, 10 APCHi females in 0 Gy, and 10 APCHi females in 9.5 Gy. Brackets indicate significant differences between 0 Gy and 9.5 Gy.

Echocardiography was performed to measure cardiac dimensions and function at 3 and 6 months after irradiation. At 3 months, radiation caused a significant increase in cardiac diameter, left ventricular inner diameter, and volume only in the APCHi males, while the wild-type female mice experienced decreases. At 6 months, these parameters were increased by radiation at end of systole in males of both genotypes (**S3–S5 Figs**), resulting in a decrease in ejection fraction and fractional shortening in these animals (**Fig 1**). On the other hand, an increase in the ratio of mitral valve E to A velocities, suggestive of a change in diastolic function, was observed in female mice. Genotype did not significantly affect the increase in mitral valve E/A in females (**S6 Fig**). Lastly, wild-type males showed a radiation-induced decrease in left ventricular posterior wall thickness at 3 and 6 months, while APCHi males were protected from this effect at 6 months (**S7 Fig**).

ECG were obtained from unanesthetized female mice at 6 months after irradiation. No differences were found in heart rate (wild-type 0 Gy: 705±18 bpm, *n* = 8; wild-type 9.5 Gy: 713±18 bpm, *n* = 4; APCHi 0 Gy: 711±18 bpm, *n* = 7; APCHi 9.5 Gy: 702±18, *n* = 5). Individual recordings were assessed and no irregularities in heart rhythm were seen.

After completion of ultrasonography and ECG measurements, hearts were collected and weighed before processing. Except for a small increase in heart weight in irradiated wild-type females, no effects of radiation or APCHi genotype were observed (**S8 Fig**).

#### Cardiac extracellular matrix

**Fig 2:**
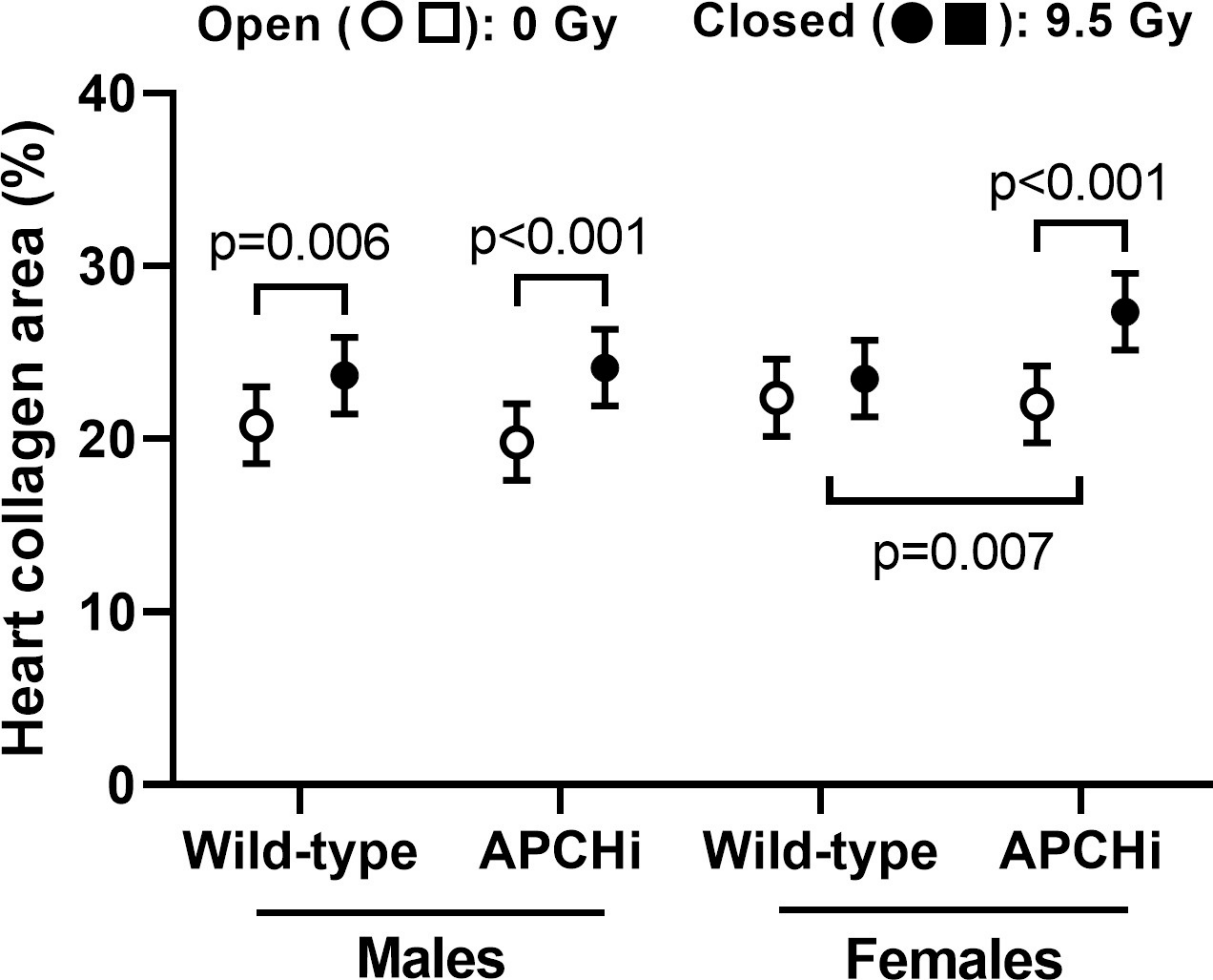
Percent of cardiac tissue area occupied by collagens at 6 months after irradiation. Means and SD of the statistical model are shown; *n* = 9 wild-type males in 0 Gy, 12 wild-type males in 9.5 Gy, 8 APCHi males in 0 Gy, 10 APCHi males in 9.5 Gy, 8 wild-type females in 0 Gy, 8 wild-type females in 9.5 Gy, 10 APCHi females in 0 Gy, and 10 APCHi females in 9.5 Gy. Short brackets indicate significant differences between 0 Gy and 9.5 Gy. Wider brackets indicate that the effect of radiation in wild-type mice is significantly different from the effect of radiation in APCHi mice.

In males, a single dose of 9.5 Gy caused an increase in collagen deposition in the hearts of both APCHi and wild-type mice. In females, the radiation-induced increase in APCHi mice was larger than that in wild-type mice (**Fig 2**). Since myofibroblasts are responsible for deposition of collagens in the heart, we examined the expression of α-SMC actin, a marker of myofibroblasts. In immunoblot analysis, a radiation-induced increase in left ventricular expression of α-SMC actin in female APCHi mice exceeded the effect of radiation in wild-type mice (**S9 Fig**), which would be in line with the finding of more radiation fibrosis in the APCHi females. However, since immunoblot analysis will detect α-SMC actin in both myofibroblasts and SMC, we also performed immunohistochemistry. As expected, more α-SMC actin positive myofibroblasts were seen in the irradiated hearts. In contrast to the immunoblot data, the total number of α-SMC actin positive myofibroblasts in immunohistochemistry seemed most pronounced in APCHi males (**S10 Fig**). The cause of the small difference in radiation fibrosis between male and female APCHi mice still needs to be determined.

After high doses of X-rays, cardiac mast cell numbers correlate with collagen deposition in the heart [13, 28]. We therefore assessed mast cell numbers as a potential indicator of adverse tissue remodeling. In contrast to our expectation, radiation decreased mast cell numbers from sham levels in all mice, regardless of sex or genotype (**S11 Fig**).

#### Cardiac immune cell infiltration

**Fig 3:**
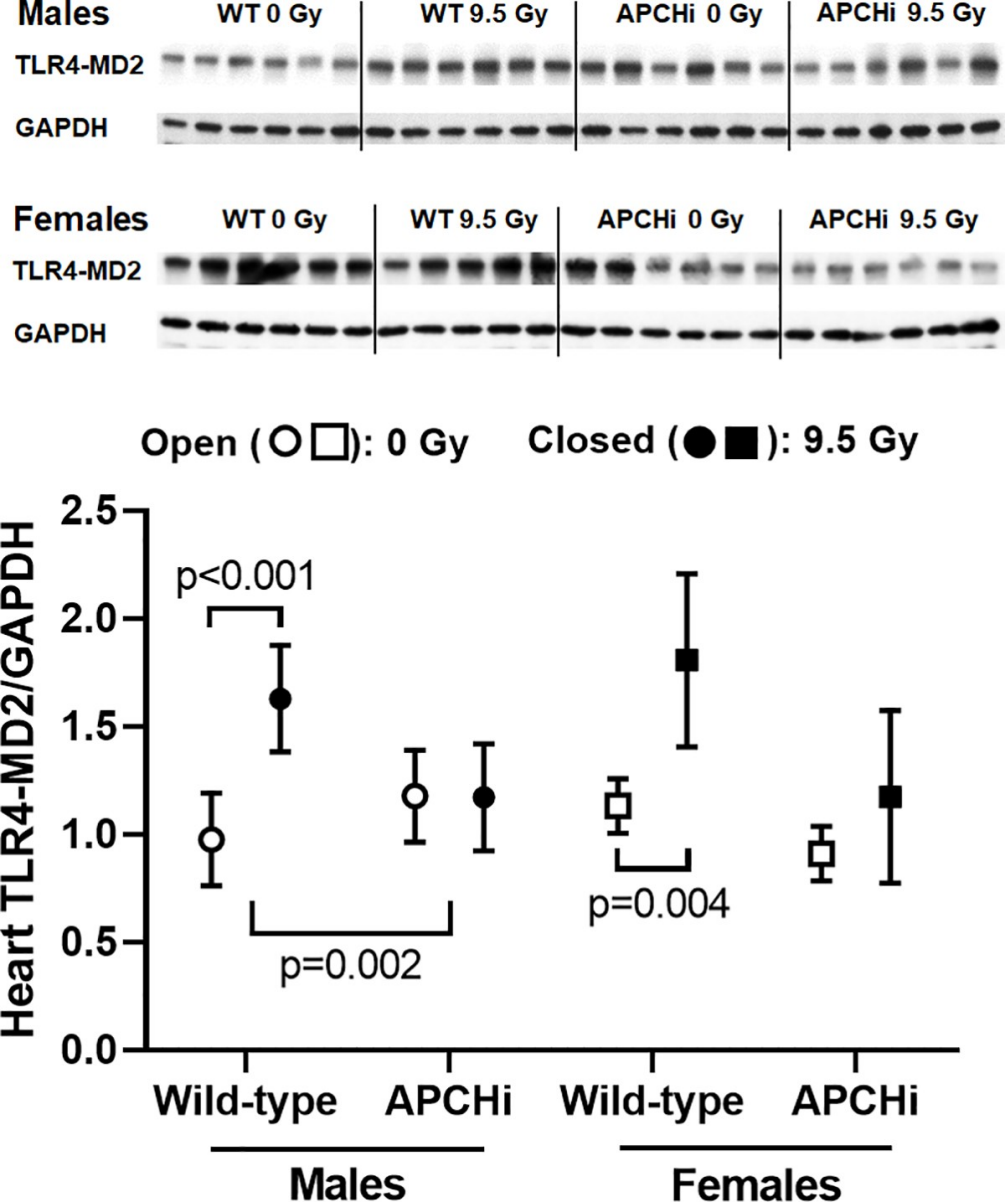
Left ventricular expression of TLR4 at 6 months after irradiation. Immunoblotting was used to assess left ventricular levels of TLR4 corrected by loading control GAPDH. Graphs indicate means and SD of the statistical model; *n* = 6 mice per group. Short brackets indicate significant differences between 0 Gy and 9.5 Gy. The wider bracket indicates that the effect of radiation in wild-type mice is significantly different from the effect of radiation in APCHi mice.

**Fig 4:**
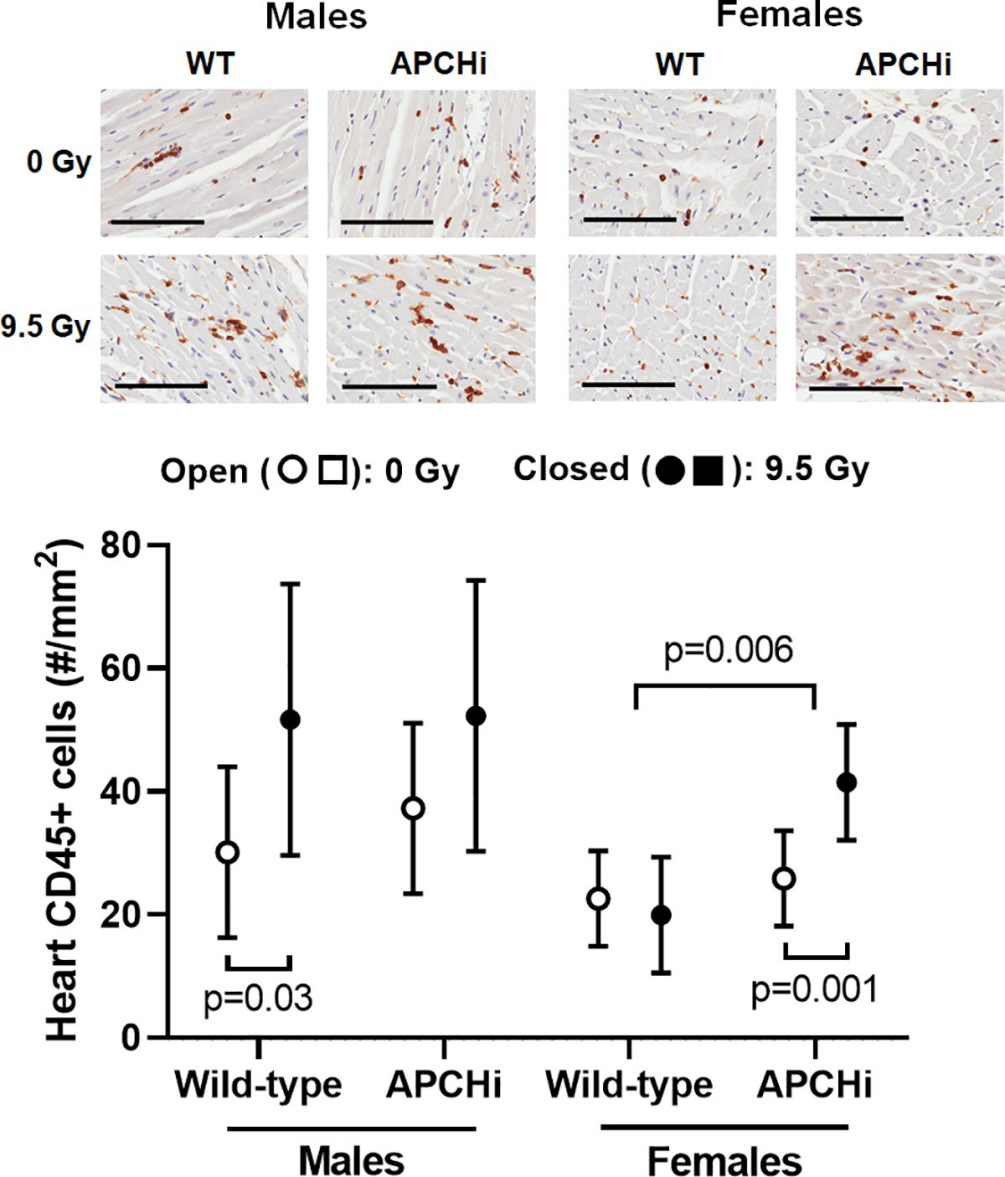
Cardiac numbers of CD45 positive cells at 6 months after irradiation. Immunohistochemistry was used to visualize CD45 positive cells in the heart. Cell counts were obtained in both left and right ventricle and divided by total tissue area. Scale bars: 100 μm. Graph indicate means and SD of the statistical model; *n* = 7 wild-type males in 0 Gy, 8 wild-type males in 9.5 Gy, 8 APCHi males in 0 Gy, 8 APCHi males in 9.5 Gy, 8 wild-type females in 0 Gy, 8 wild-type females in 9.5 Gy, 8 APCHi females in 0 Gy, and 8 APCHi females in 9.5 Gy. Short brackets indicate significant differences between 0 Gy and 9.5 Gy. The wider bracket indicates that the effect of radiation in wild-type mice is significantly different from the effect of radiation in APCHi mice.

Of all toll-like receptors (TLR) in the human and mouse heart, expression of TLR4 is most prominent [29]. TLR4 may play a role in myocardial inflammation [30–32]. Of all cardiac parameters measured, an increase in left ventricular TLR4 expression was the only outcome of radiation for which both male and female APCHi mice were protected (**Fig 3**). However, TLR4 expression did not relate to immune cell infiltration, as assessed by the number of CD45 positive cells. Radiation caused an increase in CD45 positive cells in male wild-type mice, while the effect of radiation in females was most pronounced in the APCHi mice (**Fig 4**).

#### Cardiac endothelial cells

**Fig 5:**
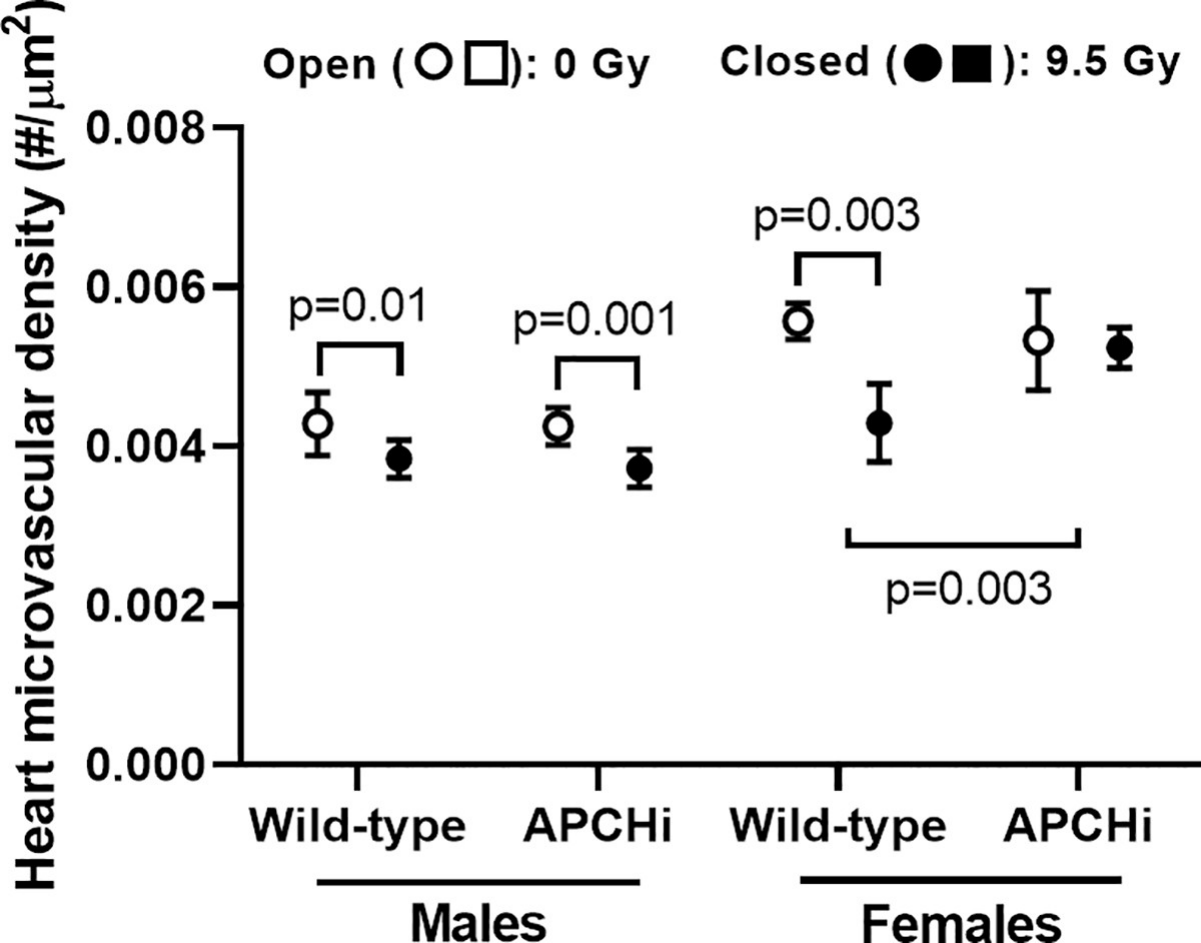
Heart microvascular density at 6 months after irradiation. Lectin staining was used to visualize endothelial cells, and the number of capillaries in both left and right ventricles was determined per tissue area. Graphs indicate means and SD of the statistical model; *n* = 9 wild-type males in 0 Gy, 8 wild-type males in 9.5 Gy, 7 APCHi males in 0 Gy, 7 APCHi males in 9.5 Gy, 8 wild-type females in 0 Gy, 5 wild-type females in 9.5 Gy, 9 APCHi females in 0 Gy, and 8 APCHi females in 9.5 Gy. Short brackets indicate significant differences between 0 Gy and 9.5 Gy. The wider bracket indicates that the effect of radiation in wild-type mice is significantly different from the effect of radiation in APCHi mice.

Ionizing radiation reduces microvascular density in the heart [6, 33]. In the current study, these radiation effects were seen in males from both genotypes; however APCHi female mice were protected from a radiation-induced loss of microvascular density (**Fig 5**). In the normal heart, von Willebrand factor (vWf) is expressed only on endothelial cells in larger arteries. After irradiation, microvascular endothelial cells express vWf as an indicator of endothelial dysfunction [14]. A single dose of 9.5 Gy caused an increase in vWf on microvascular endothelial cells in both males and females, but no clear difference was seen between the genotypes (**S12 Fig**).

### Effects of radiation in the small intestine

**Fig 6:**
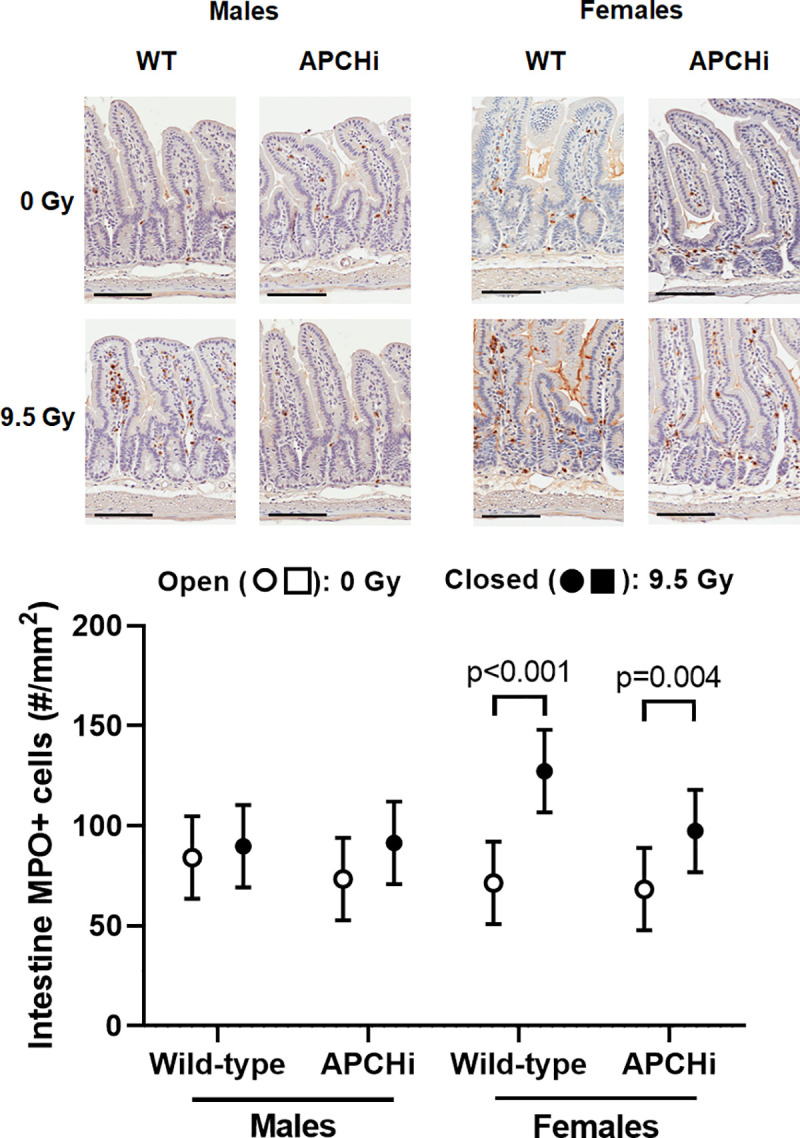
MPO-positive cells in the small intestine at 6 months after irradiation. Immunohistochemistry was used to visualize MPO-positive cells, and their numbers were determined per tissue area. Scale bars: 100 μm. Graphs indicate means and SD of the statistical model; *n* = 7 wild-type males in 0 Gy, 12 wild-type males in 9.5 Gy, 8 APCHi males in 0 Gy, 10 APCHi males in 9.5 Gy, 8 wild-type females in 0 Gy, 5 wild-type females in 9.5 Gy, 10 APCHi females in 0 Gy, and 8 APCHi females in 9.5 Gy. Brackets indicate significant differences between 0 Gy and 9.5 Gy.

**Fig 7:**
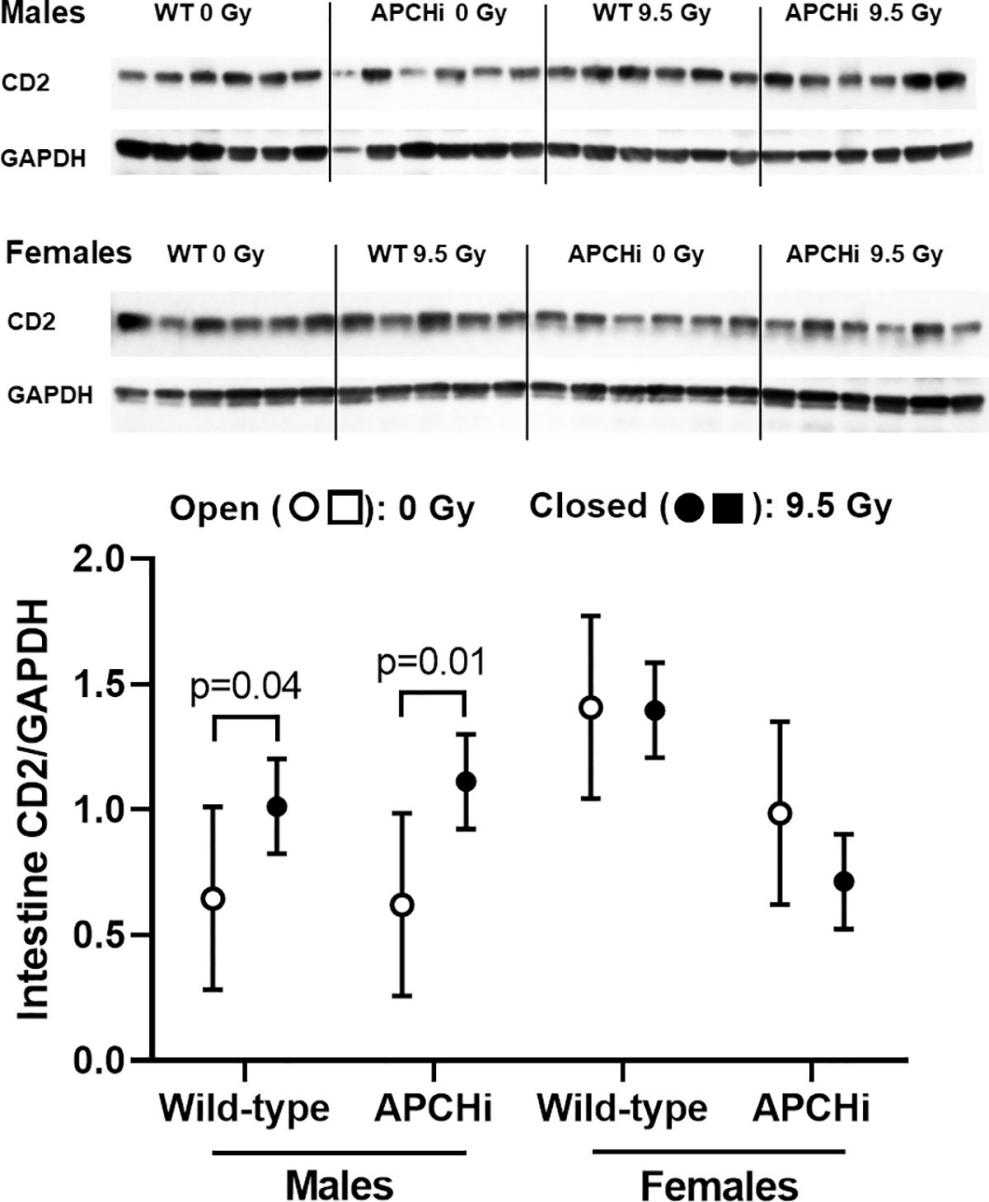
CD2 expression in the small intestinal wall at 6 months after irradiation. Immunoblotting was used to determine levels of CD2 corrected for loading control GAPDH. Graphs indicate means and SD of the statistical model; *n* = 5–6 mice per group. Brackets indicate significant differences between 0 Gy and 9.5 Gy.

At 6 months after irradiation, jejunums were collected and processed for histology and immunohistochemistry. Effects of radiation on indicators of immune cell infiltration into the intestinal wall depended upon sex. Radiation caused an increase in MPO positive cells (activated neutrophils) in females, but the increases did not significantly differ between the genotypes (**Fig 6**). Increased expression of the T-lymphocyte marker CD2, on the other hand, was induced by radiation in the intestine of male mice, again with no difference in radiation effects between the two genotypes (**Fig 7**). Lastly, radiation had only minor effects on collagen deposition, expression of α-SMC actin, and mast cell numbers in the intestinal wall (**S13 and S14 Figs**).

### Effects of radiation in the epidermis

**Fig 8:**
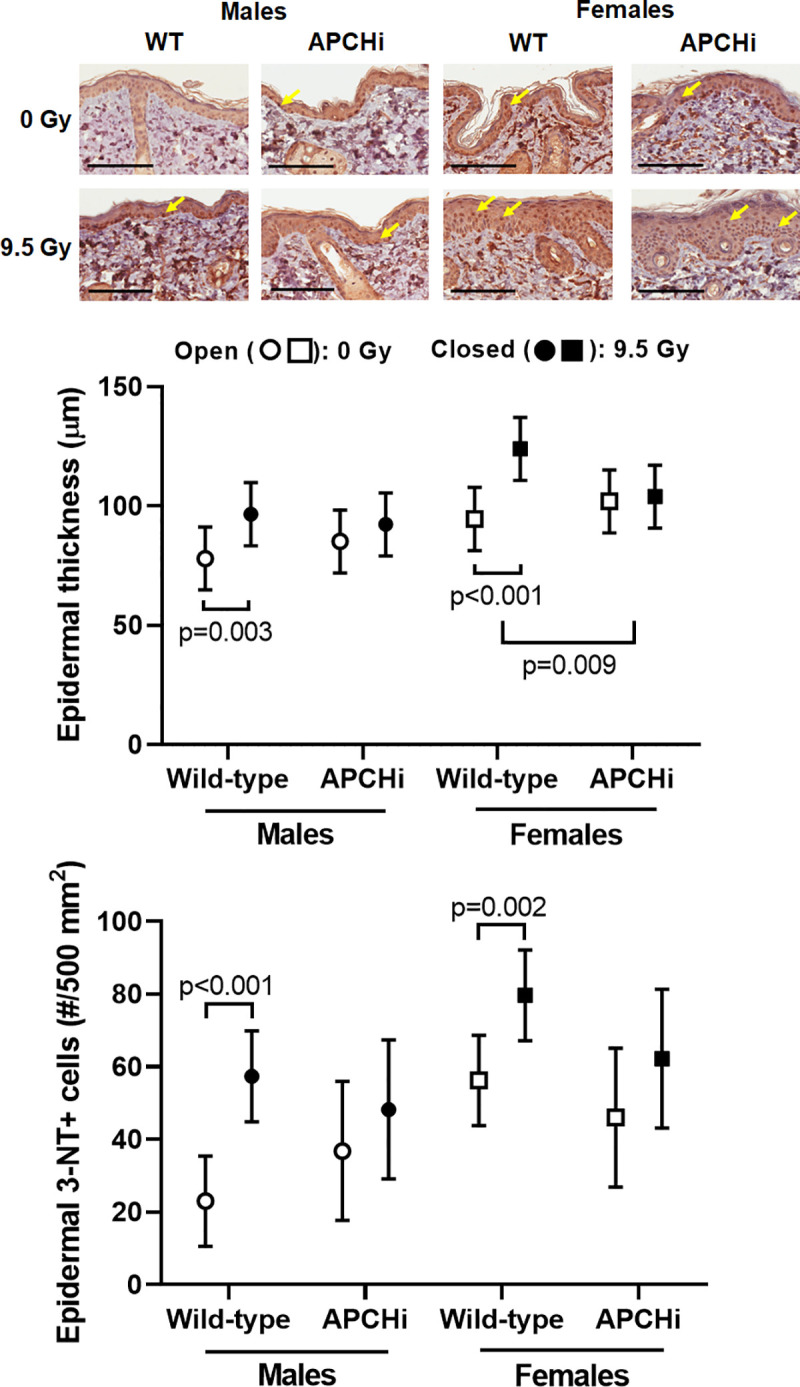
Epidermal thickness and number of 3-NT positive cells in the epidermis. Epidermal thickness was assessed in Hematoxylin & Eosin stained sections. Immunohistochemistry was used to detect 3-NT positive cells. 3-NT positive epidermal keratinocytes (arrows in micrographs) were counted. Scale bars: 100 μm. Graphs indicate means and SD of the statistical model; *n* = 6–9 wild-type males in 0 Gy, 10 wild-type males in 9.5 Gy, 6–8 APCHi males in 0 Gy, 8–9 APCHi males in 9.5 Gy, 7–8 wild-type females in 0 Gy, 5–6 wild-type females in 9.5 Gy, 8–9 APCHi females in 0 Gy, and 8–9 APCHi females in 9.5 Gy. The short brackets indicate a significant difference between 0 Gy and 9.5 Gy. The wider bracket indicates that the effect of radiation in wild-type mice is significantly different from the effect of radiation in APCHi mice.

At 6 months after irradiation, dorsal skin was collected and processed for histology and immunohistochemistry. The area of the epidermis positive for 4-HNE was not changed (**S15 Fig**). On the other hand, radiation caused an increase in epidermal thickness and the number of 3-NT positive epidermal keratinocytes of both male and female wild-type mice, and the APCHi females were protected from radiation-induced increases in epidermal thickness (**Fig 8**).

### Multiple comparisons and tests

We examined 26 outcomes that were observed at only one time point (e.g., 6 months), and 18 outcomes observed at 3 and 6 months. For each outcome at each time point, we conducted 3 tests in the males and in the females: the radiation effects within each genotype (i.e., 9.5 Gy vs sham) and the radiation effects between the genotypes (i.e., the interaction). Altogether there were 372 tests, of which 111 had *p* ≤ 0.05. The positive False Discovery Rate was estimated to be 0.066; that is, about 7 of the significant results may be false discoveries, with a 95% confidence upper bound of 12 false discoveries.

## Discussion

APC is a critical component in the circulation with anti-coagulant, anti-inflammatory and cytoprotective properties [15–18]. We have previously shown that recombinant APC is an effective mitigator of acute radiation injury when administered to male C57BL/6 mice in two injections at 24 and 48 hours after exposure to a single dose of 9.5 Gy total body γ-rays [19]. However, the role of APC in long-term radiation toxicity is largely unknown. The current study used a mouse model that expresses the D168F/N173K mouse analogue of the hyperactivatable human D167F/D172K protein C variant [20], resulting in 2- to 3-fold increased plasma levels of murine APC [21]. To study the effects of increased APC on DEARE in 3 organ systems, we exposed mice to 9.5 Gy γ-rays while their hind-legs were shielded and followed them for 6 months to examine the heart, small intestine and skin. A graphic summary of the research results is provided in **S16 Fig**.

The dose of 9.5 Gy was selected to be consistent with our prior study of APC administration in total body irradiation, in which two bolus injections of mouse wild-type APC improved 30-day survival [19]. On the other hand, we do not see a radiation survival benefit in APCHi mice compared to wild-type mice in the current study. Since APCs have a short plasma half-life [34, 35], plasma concentrations and duration of increased plasma levels after a bolus administration of APC will be very different from a continued upregulation of APC as studied with a genetic approach. The effects of APC administration on DEARE still need to be investigated.

In the model used in this study, the hind-legs of the mice are shielded from irradiation to protect a portion of the bone marrow and allow long-term survival of the animals. Therefore, a shortcoming of this model is that the mice do not experience H-ARS before they mature into the phase of DEARE. In a prior study with total body irradiation with single doses of 8.5–8.7 Gy γ-rays in male and female C57BL/6 mice, Unthank et al identified injury and fibrosis in the heart [4]. However, these effects were seen at 9–21 months after irradiation. We therefore expect only small effects of a single dose of 9.5 Gy when measured at 6 months. Nonetheless, an improvement or worsening of DEARE by the APCHi genotype may still be detected.

APC has anti-inflammatory properties via modulation of endothelial cell function and signaling pathways in immune cells [36, 37]. A significant upregulation of TLR4 by radiation in the heart was only seen in the wild-type male and female mice. While the role of TLR4 in the irradiated heart is not yet fully understood, prior studies have shown a role for TLR4 in myocardial inflammation due to myocardial infarction [28], myocarditis [29], and in heart failure [30]. However, in APCHi female mice, the reduced TLR4 expression did not translate into a protection against a cardiac infiltration of immune cells. In addition, there was no effect of high APC on intestinal inflammatory cells, as measured by MPO staining and CD2 expression. This is in contrast to the beneficial effects of thrombomodulin administration or thrombomodulin upregulation in intestinal radiation injury [38, 39].

APC interacts with several known and putative endothelial cell receptors [40–42]. Through interaction with these receptors, APC inhibits endothelial cell death and senescence [15]. APCHi female mice were protected from a radiation-induced loss of microvascular density in the heart, but there were no obvious differences in the expression of endothelial injury marker vWf. More research is needed to determine how APC affects endothelial and vascular function in irradiated organs and tissues.

APC has been shown to reduce oxidative stress in various disease models [43–45]. This may explain the reduced number of 3-NT positive cells in the epidermis of irradiated APCHi mice. In addition to its effects on endothelial and immune cells, APC inhibits cell death and promotes proliferation of keratinocytes in the skin [46]. APC has also been suggested to induce anti-inflammatory and barrier protective effects by modulating a number of proteases and cytokines in keratinocytes and the skin [47–50]. The anti-inflammatory action of APC may explain why APCHi female mice showed reduced skin thickening upon radiation.

Altogether, in several cardiovascular outcomes and in the skin, differences were seen between males and females in their response to irradiation and in the interaction between radiation and high APC. Sex differences have been reported in adverse effects of accidental radiation exposure in human subjects [51]. A difference in cardiovascular radiation injury between males and females may not be very surprising, since cardiovascular disease risk in general depends on sex [52, 53], and heart disease presents itself differently in the two sexes [54]. Differences in radiation response between males and females may be in part explained by differences in immunity and immune cell function [55, 56]. Moreover, male and female cells have been speculated to respond differently to oxidative and proteolytic stress [57], and sex hormones have been thought to play a role in the development of organ fibrosis [58, 59].

In addition, there are some known differences in the protein C pathway between male and female human subjects that may help explain differences in the effects of high APC between male and female mice. Healthy female subjects show higher rates of thrombin generation compared to males [60]. Moreover, female subjects may have a slightly higher resistance to APC anticoagulant activity [61]. Males and females can also differ in the expression of receptors that are part of the protein C pathway, such as suggested by a difference in plasma levels of soluble endothelial protein C receptor [62], or the response to the activation of proteinase-activated receptor-1, one of the main receptors that mediates the effects of APC on endothelial and epithelial cells [63].

In conclusion, prolonged high levels of APC in a transgenic mouse model had little effects on indicators of DEARE in the heart, small intestine and skin. In both sexes, high APC protected only against an increase in TLR4 expression in the heart, and epidermal thickening and the number of 3-NT positive epidermal keratinocytes in the skin. Additional studies will be required to test the administration of APC as a mitigator of late radiation injuries and identify biological mechanisms by which APC may have different effects on certain DEARE in males compared to females.

## Supporting information

S1 Fig Thirty-day survival after 9.5 Gy total body irradiation. *n* = 7 wild-type males, 10 APCHi males, 8 wild-type females, and 10 APCHi females. No differences were seen between the genotypes in both sexes.
(PDF)

S2 Fig Bodyweight at 3 and 6 months after irradiation. Means and standard deviations (SD) of the statistical model are shown; *n* = 9 wild-type males in 0 Gy, 12 wild-type males in 9.5 Gy, 8 APCHi males in 0 Gy, 10 APCHi males in 9.5 Gy, 8 wild-type females in 0 Gy, 8 wild-type females in 9.5 Gy, 10 APCHi females in 0 Gy, and 10 APCHi females in 9.5 Gy. Short brackets indicate significant differences between 0 Gy and 9.5 Gy. The wider bracket indicates that the effect of radiation in wild-type mice is significantly different from the effect of radiation in APCHi mice.
(PDF)

S3 Fig Cardiac diameter as measured with echocardiography at 3 and 6 months after irradiation. Means and SD of the statistical model are shown; *n* = 9 wild-type males in 0 Gy, 12 wild-type males in 9.5 Gy, 8 APCHi males in 0 Gy, 10 APCHi males in 9.5 Gy, 8 wild-type females in 0 Gy, 8 wild-type females in 9.5 Gy, 10 APCHi females in 0 Gy, and 10 APCHi females in 9.5 Gy. Short brackets indicate significant differences between 0 Gy and 9.5 Gy. Wider brackets indicates that the effect of radiation in wild-type mice is significantly different from the effect of radiation in APCHi mice.
(PDF)

S4 Fig Left ventricular inner diameter (LVID) as measured with echocardiography at 3 and 6 months after irradiation. Means and SD of the statistical model are shown; *n* = 9 wild-type males in 0 Gy, 12 wild-type males in 9.5 Gy, 8 APCHi males in 0 Gy, 10 APCHi males in 9.5 Gy, 8 wild-type females in 0 Gy, 8 wild-type females in 9.5 Gy, 10 APCHi females in 0 Gy, and 10 APCHi females in 9.5 Gy. Short brackets indicate significant differences between 0 Gy and 9.5 Gy. The wider bracket indicates that the effect of radiation in wild-type mice is significantly different from the effect of radiation in APCHi mice.
(PDF)

S5 Fig Left ventricular volume as measured with echocardiography at 3 and 6 months after irradiation. Means and SD of the statistical model are shown; *n* = 9 wild-type males in 0 Gy, 12 wild-type males in 9.5 Gy, 8 APCHi males in 0 Gy, 10 APCHi males in 9.5 Gy, 8 wild-type females in 0 Gy, 8 wild-type females in 9.5 Gy, 10 APCHi females in 0 Gy, and 10 APCHi females in 9.5 Gy. Short brackets indicate significant differences between 0 Gy and 9.5 Gy. Wider brackets indicates that the effect of radiation in wild-type mice is significantly different from the effect of radiation in APCHi mice.
(PDF)

S6 Fig Ratio of mitral valve E to A velocities as measured with echocardiography at 3 and 6 months after irradiation. Means and SD of the statistical model are shown; *n* = 9 wild-type males in 0 Gy, 12 wild-type males in 9.5 Gy, 8 APCHi males in 0 Gy, 10 APCHi males in 9.5 Gy, 8 wild-type females in 0 Gy, 8 wild-type females in 9.5 Gy, 10 APCHi females in 0 Gy, and 10 APCHi females in 9.5 Gy. Brackets indicate significant differences between 0 Gy and 9.5 Gy.
(PDF)

S7 Fig Left ventricular posterior wall thickness (LVPW) as measured with echocardiography at 3 and 6 months after irradiation. Means and SD of the statistical model are shown; *n* = 9 wild-type males in 0 Gy, 12 wild-type males in 9.5 Gy, 8 APCHi males in 0 Gy, 10 APCHi males in 9.5 Gy, 8 wild-type females in 0 Gy, 8 wild-type females in 9.5 Gy, 10 APCHi females in 0 Gy, and 10 APCHi females in 9.5 Gy. Short brackets indicate significant differences between 0 Gy and 9.5 Gy. Wider brackets indicates that the effect of radiation in wild-type mice is significantly different from the effect of radiation in APCHi mice.
(PDF)

S8 Fig Heart weight divided by body weight at 6 months after irradiation. Means and SD of the statistical model are shown; *n* = 9 wild-type males in 0 Gy, 12 wild-type males in 9.5 Gy, 8 APCHi males in 0 Gy, 10 APCHi males in 9.5 Gy, 8 wild-type females in 0 Gy, 8 wild-type females in 9.5 Gy, 10 APCHi females in 0 Gy, and 10 APCHi females in 9.5 Gy. The bracket indicates significant differences between 0 Gy and 9.5 Gy.
(PDF)

S9 Fig Expression of α-smooth muscle cell (α-SMC) actin in the heart at 6 months after irradiation. Immunoblotting to determine left ventricular levels of α-SMC actin corrected for the loading control GAPDH. Means and SD of the statistical model are shown; *n* = 6 mice per group. The short bracket indicates a significant difference between 0 Gy and 9.5 Gy. The wider bracket indicates that the effect of radiation in wild-type mice is significantly different from the effect of radiation in APCHi mice.
(PDF)

S10 Fig Expression of α-SMC actin in the heart at 6 months after irradiation. Immunohistochemistry was used to assess α-SMC actin expression in the whole heart. Scale bar = 100 μm. In addition to vascular smooth muscle cells (red arrows), spindle-shaped α-SMC actin positive cells (green arrows) were identified throughout the myocardium. These cells are likely myofibroblasts. Each stained slide was scored on a scale from 0 (no α-SMC actin positive myofibroblasts) to 4 (more than 100 SMC actin positive myofibroblasts); *n* = 7–8 mice per group. Individual scores are listed in the table.
(PDF)

S11 Fig Mast cell numbers in the heart at 6 months after irradiation. Toluidine Blue staining was used to visualize mast cells (scale bar = 100 μm), and mast cell numbers in both left and right ventricles were determined. Means and SD of the statistical model are shown. *n* = 8–10 animals per group. Brackets indicate significant differences between 0 Gy and 9.5 Gy.
(PDF)

S12 Fig Von Willebrand factor (vWf) staining in the heart. Immunohistochemistry was used to assess vWf expression in the heart. In the normal heart, vWf is expressed only on endothelial cells in larger arteries. After irradiation, vWf staining is seen on microvascular endothelial cells and extracellular matrix. Scale bar = 100 μm. Heart sections stained for vWf were scored on a scale from 0 (vWf only in larger arteries) to 3 (vWf positive capillaries throughout the myocardium). The score for each animal is listed in the tables; *n* = 4–7 mice per group.
(PDF)

S13 Fig Expression of α-SMC actin and collagen deposition in the small intestinal wall at 6 months after irradiation. **A)** Immunoblotting to determine levels of α-SMC actin corrected for the loading control GAPDH. Means and SD of the statistical model are shown; *n* = 6 mice per group. **B)** Collagen deposition as measured from Sirius Red histology. Means and SD of the statistical model are shown; *n* = 8 wild-type males in 0 Gy, 12 wild-type males in 9.5 Gy, 8 APCHi males in 0 Gy, 10 APCHi males in 9.5 Gy, 8 wild-type females in 0 Gy, 5 wild-type females in 9.5 Gy, 10 APCHi females in 0 Gy, and 9 APCHi females in 9.5 Gy. The short bracket indicates a significant difference between 0 Gy and 9.5 Gy. The wider bracket indicates that the effect of radiation in wild-type mice is significantly different from the effect of radiation in APCHi mice.
(PDF)

S14 Fig Mast cell numbers in the intestinal wall at 6 months after irradiation. Mast cell numbers as determined from Toluidine Blue histology. Means and SD of the statistical model are shown; *n* = 8 wild-type males in 0 Gy, 12 wild-type males in 9.5 Gy, 8 APCHi males in 0 Gy, 10 APCHi males in 9.5 Gy, 8 wild-type females in 0 Gy, 5 wild-type females in 9.5 Gy, 10 APCHi females in 0 Gy, and 9 APCHi females in 9.5 Gy. The bracket indicates a significant difference between 0 Gy and 9.5 Gy.
(PDF)

S15 Fig 4-HNE immunostaining in the epidermis. Positivity for 4-HNE was assessed with immunohistochemistry. Graphs indicate means and SD of the statistical model; *n* = 6–9 wild-type males in 0 Gy, 10 wild-type males in 9.5 Gy, 6–8 APCHi males in 0 Gy, 8–9 APCHi males in 9.5 Gy, 7–8 wild-type females in 0 Gy, 5–6 wild-type females in 9.5 Gy, 8–9 APCHi females in 0 Gy, and 8–9 APCHi females in 9.5 Gy.
(PDF)

S16 Fig Graphic summary of research results obtained from male and female wild-type and APCHi mice at 6 months after 9.5 Gy compared to time-matched sham-irradiated controls. Arrow up: increase in irradiated animals compared to sham controls within the same genotype. Arrow down: decrease in irradiated animals compared to sham controls within the same genotype. Horizontal line: no significant effect of radiation. Shaded areas highlight differences in radiation response between the two genotypes.
(PDF)

S1 Table Primary antibodies in immunohistochemistry and immunoblotting. (PDF)

S2 Table Power analyses: Effect sizes detectable with 0.80 power on a 0.05 significance level. The table shows detectable effect sizes, based on two sample sizes (*n* = 6 and *n* = 15) in each of the radiation×genotype×sex groups.
(PDF)

S1 Data APCHi full dataset. (XLSX)

S1 Raw images (PDF)
