# Supplementary material for: Sex-dependent effects of genetic upregulation of activated protein C on delayed effects of acute radiation exposure in the mouse heart, small intestine, and skin
Source: PLoS One. 2021 May 24;16(5):e0252142. doi: 10.1371/journal.pone.0252142 (PMC8143413; doi:10.1371/journal.pone.0252142)
Supplement: S1 Fig — n = 7 wild-type males, 10 APCHi males, 8 wild-type females, and 10 APCHi females. No differences were seen between the genotypes in both sexes. (PDF) [file pone.0252142.s001.pdf]

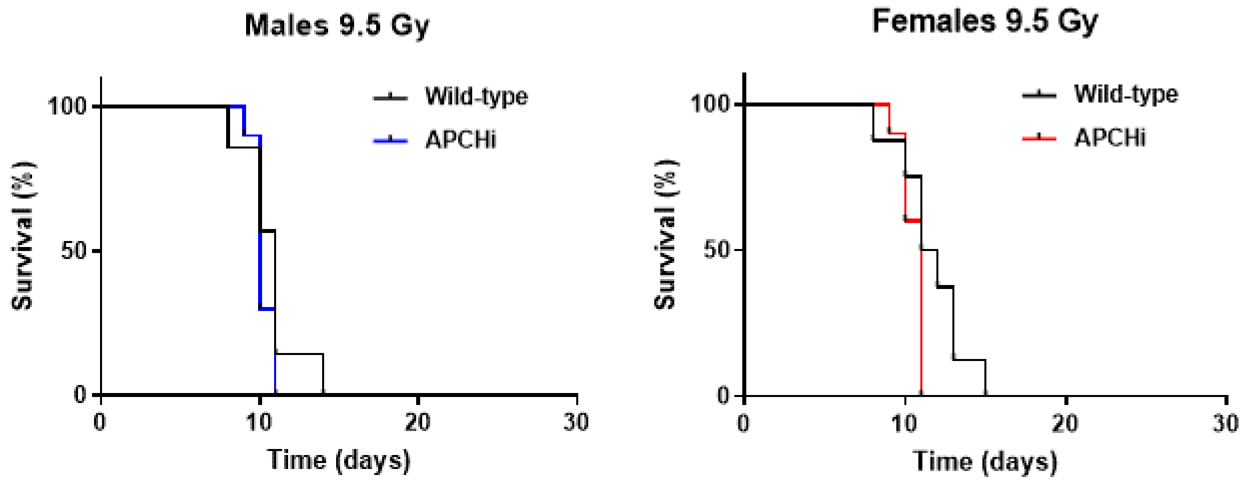

S1 Fig. Thirty-day survival after 9.5 Gy total body irradiation. *n*=7 wild-type males, 10 APCHi males, 8 wild-type females, and 10 APCHi females. No differences were seen between the genotypes in both sexes.
