# Supplementary material for: Sex-dependent effects of genetic upregulation of activated protein C on delayed effects of acute radiation exposure in the mouse heart, small intestine, and skin
Source: PLoS One. 2021 May 24;16(5):e0252142. doi: 10.1371/journal.pone.0252142 (PMC8143413; doi:10.1371/journal.pone.0252142)
Supplement: S3 Fig — Means and SD of the statistical model are shown; n = 9 wild-type males in 0 Gy, 12 wild-type males in 9.5 Gy, 8 APCHi males in 0 Gy, 10 APCHi males in 9.5 Gy, 8 wild-type females in 0 Gy, 8 wild-type females in 9.5 Gy, 10 APCHi females in 0 Gy, and 10 APCHi females in 9.5 Gy. Short brackets indicate significant differences between 0 Gy and 9.5 Gy. Wider brackets indicates that the effect of radiation in wild-type mice is significantly different from the effect of radiation in APCHi mice. (PDF) [file pone.0252142.s003.pdf]

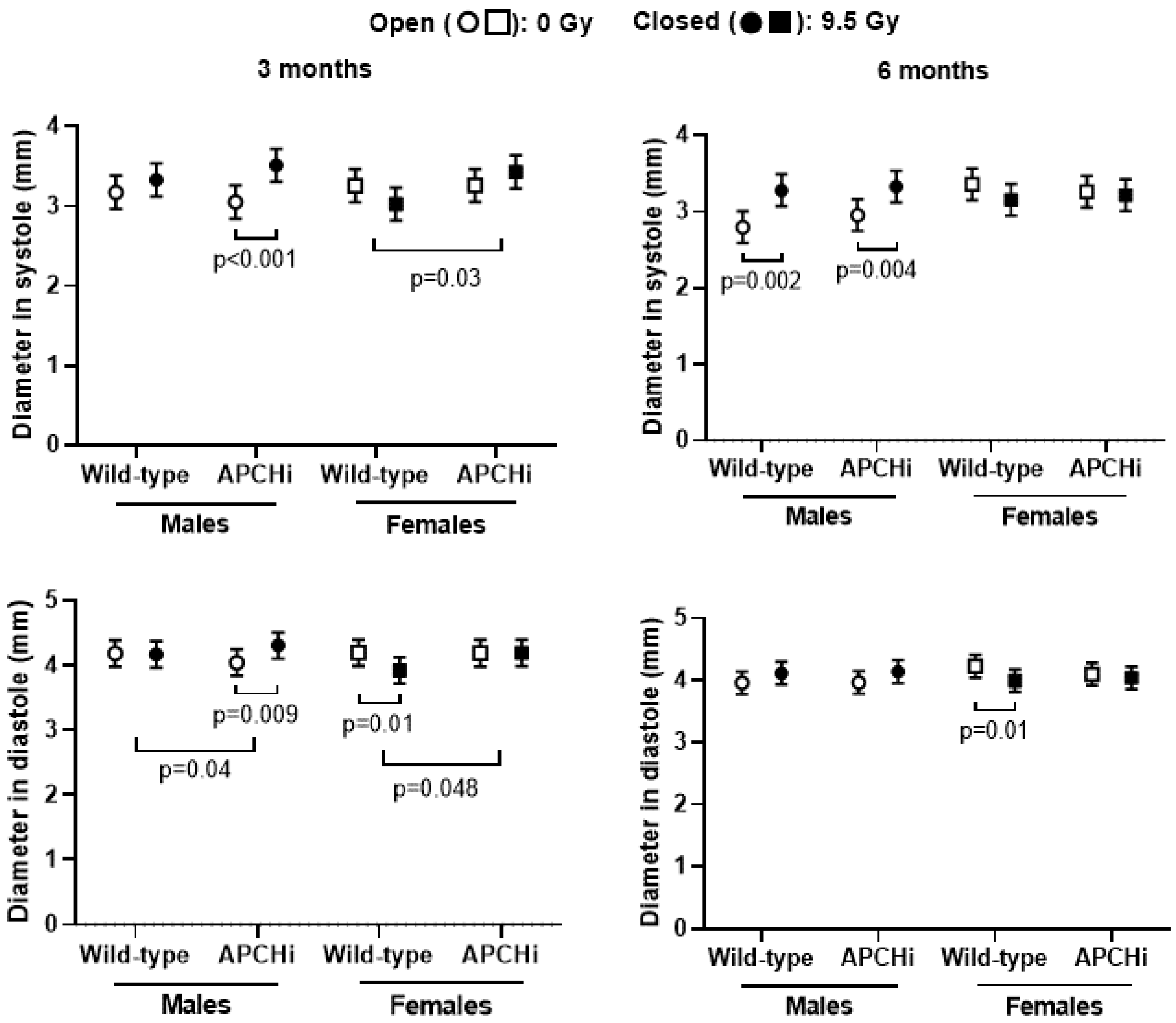

S3 Fig. Cardiac diameter as measured with echocardiography at 3 and 6 months after irradiation. Means and SD of the statistical model are shown; *n*=9 wild-type males in 0 Gy, 12 wild-type males in 9.5 Gy, 8 APCHi males in 0 Gy, 10 APCHi males in 9.5 Gy, 8 wild-type females in 0 Gy, 8 wild-type females in 9.5 Gy, 10 APCHi females in 0 Gy, and 10 APCHi females in 9.5 Gy. Short brackets indicate significant differences between 0 Gy and 9.5 Gy. Wider brackets indicates that the effect of radiation in wild-type mice is significantly different from the effect of radiation in APCHi mice.
