# Supplementary material for: Sex-dependent effects of genetic upregulation of activated protein C on delayed effects of acute radiation exposure in the mouse heart, small intestine, and skin
Source: PLoS One. 2021 May 24;16(5):e0252142. doi: 10.1371/journal.pone.0252142 (PMC8143413; doi:10.1371/journal.pone.0252142)
Supplement: S9 Fig — Immunoblotting to determine left ventricular levels of α-SMC actin corrected for the loading control GAPDH. Means and SD of the statistical model are shown; n = 6 mice per group. The short bracket indicates a significant difference between 0 Gy and 9.5 Gy. The wider bracket indicates that the effect of radiation in wild-type mice is significantly different from the effect of radiation in APCHi mice. (PDF) [file pone.0252142.s009.pdf]

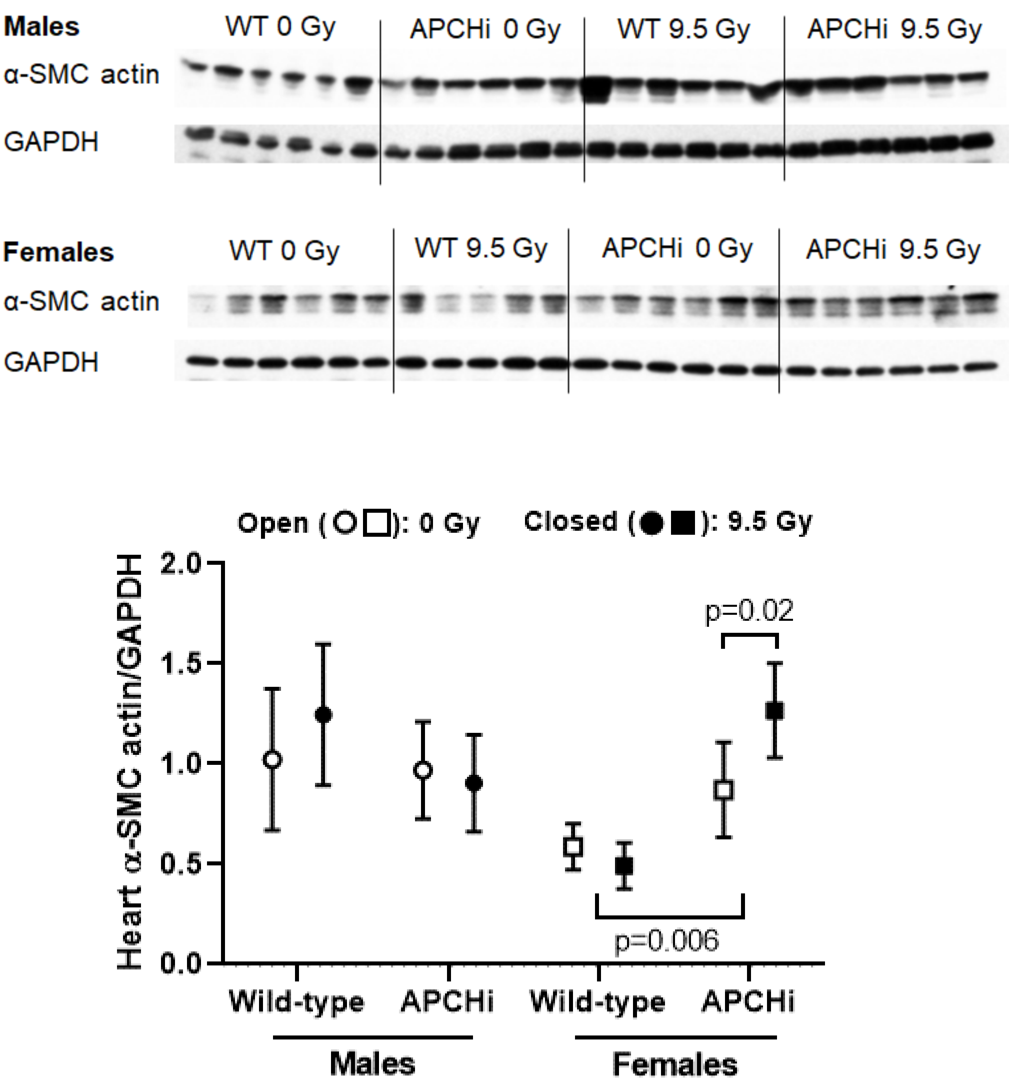

S9 Fig. Expression of α-smooth muscle cell (α-SMC) actin in the heart at 6 months after irradiation. Immunoblotting to determine left ventricular levels of α-SMC actin corrected for the loading control GAPDH. Means and SD of the statistical model are shown; *n*=6 mice per group. The short bracket indicates a significant difference between 0 Gy and 9.5 Gy. The wider bracket indicates that the effect of radiation in wild-type mice is significantly different from the effect of radiation in APCHi mice.
