# Supplementary material for: Sex-dependent effects of genetic upregulation of activated protein C on delayed effects of acute radiation exposure in the mouse heart, small intestine, and skin
Source: PLoS One. 2021 May 24;16(5):e0252142. doi: 10.1371/journal.pone.0252142 (PMC8143413; doi:10.1371/journal.pone.0252142)
Supplement: S10 Fig — Immunohistochemistry was used to assess α-SMC actin expression in the whole heart. Scale bar = 100 μm. In addition to vascular smooth muscle cells (red arrows), spindle-shaped α-SMC actin positive cells (green arrows) were identified throughout the myocardium. These cells are likely myofibroblasts. Each stained slide was scored on a scale from 0 (no α-SMC actin positive myofibroblasts) to 4 (more than 100 SMC actin positive myofibroblasts); n = 7–8 mice per group. Individual scores are listed in the table. (PDF) [file pone.0252142.s010.pdf]

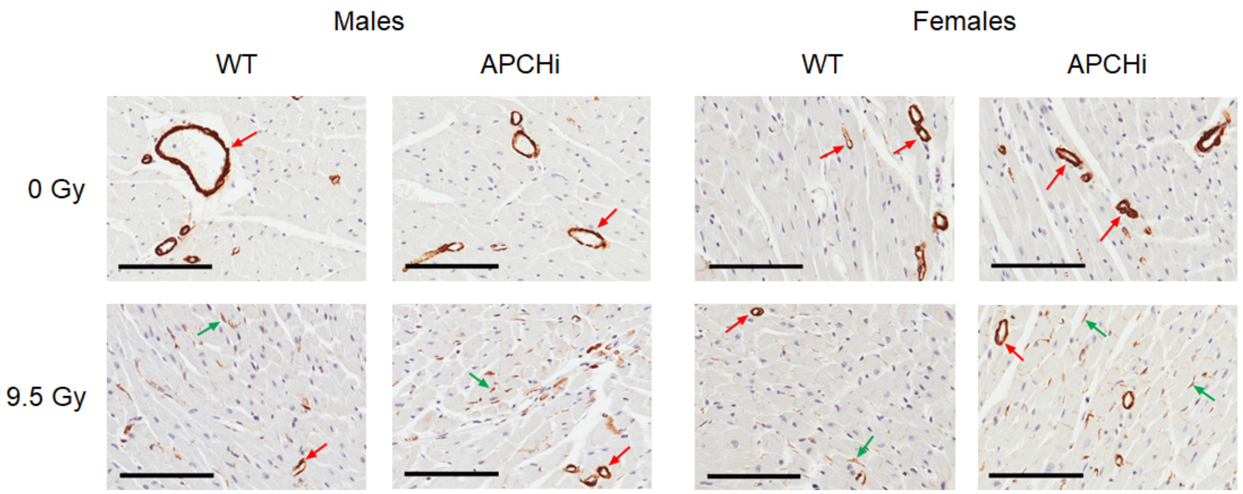

Males

**Wild-type**	**0 Gy**	0	0	0	0	0	0	0	0
**Wild-type**	**9.5 Gy**	0	0	1	1	1	1	2	2
**APCHi**	**0 Gy**	0	0	0	0	0	0	1	1
**APCHi**	**9.5 Gy**	1	1	2	2	3	4	4	

Females

**Wild-type**	**0 Gy**	0	0	0	0	1	1	1	3
**Wild-type**	**9.5 Gy**	1	2	2	2	3	3	3	
**APCHi**	**0 Gy**	0	0	0	0	1	1	1	1
**APCHi**	**9.5 Gy**	1	1	1	1	2	2	2	3

S10 Fig. Expression of α-SMC actin in the heart at 6 months after irradiation. Immunohistochemistry was used to assess α-SMC actin expression in the whole heart. Scale bar = 100 μm. In addition to vascular smooth muscle cells (red arrows), spindle-shaped α-SMC actin positive cells (green arrows) were identified throughout the myocardium. These cells are likely myofibroblasts. Each stained slide was scored on a scale from 0 (no α-SMC actin positive myofibroblasts) to 4 (more than 100 SMC actin positive myofibroblasts); *n*=7-8 mice per group. Individual scores are listed in the table.
