# Supplementary material for: Sex-dependent effects of genetic upregulation of activated protein C on delayed effects of acute radiation exposure in the mouse heart, small intestine, and skin
Source: PLoS One. 2021 May 24;16(5):e0252142. doi: 10.1371/journal.pone.0252142 (PMC8143413; doi:10.1371/journal.pone.0252142)
Supplement: S11 Fig — Toluidine Blue staining was used to visualize mast cells (scale bar = 100 μm), and mast cell numbers in both left and right ventricles were determined. Means and SD of the statistical model are shown. n = 8–10 animals per group. Brackets indicate significant differences between 0 Gy and 9.5 Gy. (PDF) [file pone.0252142.s011.pdf]

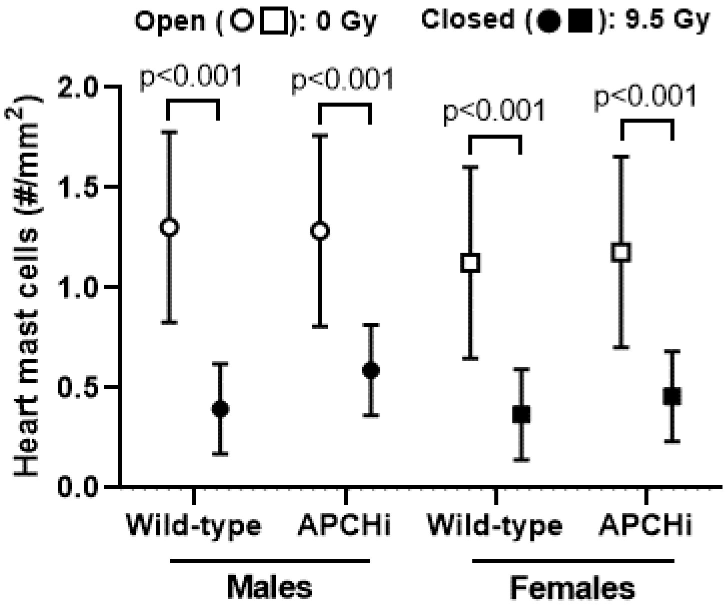

S11 Fig. Mast cell numbers in the heart at 6 months after irradiation. Toluidine Blue staining was used to visualize mast cells (scale bar = 100 µm), and mast cell numbers in both left and right ventricles were determined. Means and SD of the statistical model are shown. *n*=8-10 animals per group. Brackets indicate significant differences between 0 Gy and 9.5 Gy.
