# Supplementary material for: Sex-dependent effects of genetic upregulation of activated protein C on delayed effects of acute radiation exposure in the mouse heart, small intestine, and skin
Source: PLoS One. 2021 May 24;16(5):e0252142. doi: 10.1371/journal.pone.0252142 (PMC8143413; doi:10.1371/journal.pone.0252142)
Supplement: S12 Fig — Immunohistochemistry was used to assess vWf expression in the heart. In the normal heart, vWf is expressed only on endothelial cells in larger arteries. After irradiation, vWf staining is seen on microvascular endothelial cells and extracellular matrix. Scale bar = 100 μm. Heart sections stained for vWf were scored on a scale from 0 (vWf only in larger arteries) to 3 (vWf positive capillaries throughout the myocardium). The score for each animal is listed in the tables; n = 4–7 mice per group. (PDF) [file pone.0252142.s012.pdf]

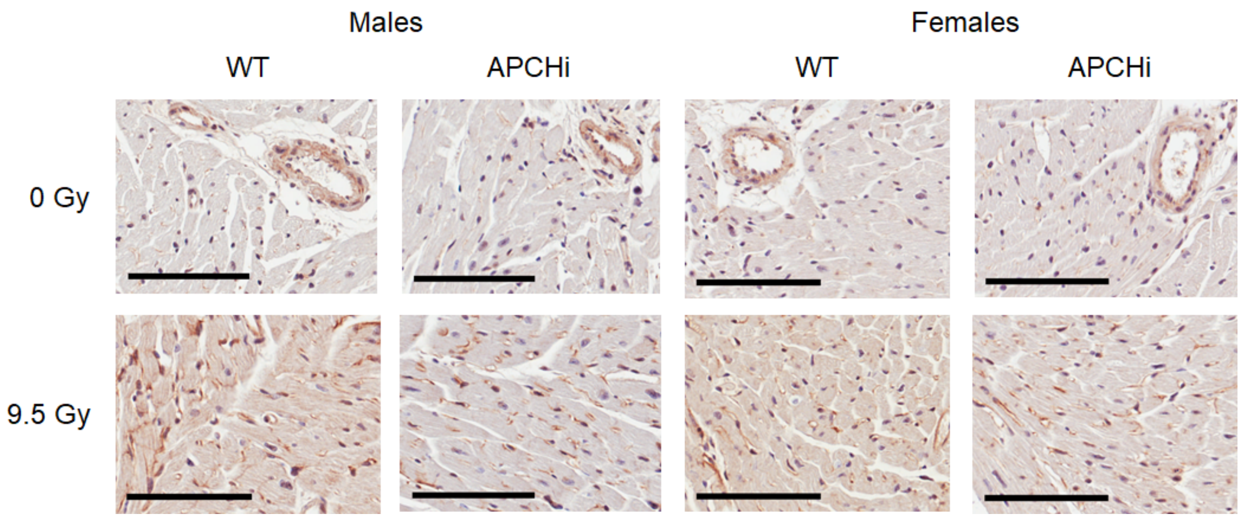

Males

**WT**	**0 Gy**	0	0	1	2			
**WT**	**9.5 Gy**	2	2	2	2	2	3	3
**APCHi**	**0 Gy**	0	0	1	2			
**APCHi**	**9.5 Gy**	1	2	2	2	3	3	

Females

**WT**	**0 Gy**	0	1	1	1	1	1
**WT**	**9.5 Gy**	0	1	1	2	3	
**APCHi**	**0 Gy**	0	0	0	0	1	1
**APCHi**	**9.5 Gy**	0	1	2	2	2	3

S12 Fig. Von Willebrand factor (vWf) staining in the heart. Immunohistochemistry was used to assess vWf expression in the heart. In the normal heart, vWf is expressed only on endothelial cells in larger arteries. After irradiation, vWf staining is seen on microvascular endothelial cells and extracellular matrix. Scale bar = 100 µm. Heart sections stained for vWf were scored on a scale from 0 (vWf only in larger arteries) to 3 (vWf positive capillaries throughout the myocardium). The score for each animal is listed in the tables; *n*=4-7 mice per group.
