# Supplementary material for: Sex-dependent effects of genetic upregulation of activated protein C on delayed effects of acute radiation exposure in the mouse heart, small intestine, and skin
Source: PLoS One. 2021 May 24;16(5):e0252142. doi: 10.1371/journal.pone.0252142 (PMC8143413; doi:10.1371/journal.pone.0252142)
Supplement: S14 Fig — Mast cell numbers as determined from Toluidine Blue histology. Means and SD of the statistical model are shown; n = 8 wild-type males in 0 Gy, 12 wild-type males in 9.5 Gy, 8 APCHi males in 0 Gy, 10 APCHi males in 9.5 Gy, 8 wild-type females in 0 Gy, 5 wild-type females in 9.5 Gy, 10 APCHi females in 0 Gy, and 9 APCHi females in 9.5 Gy. The bracket indicates a significant difference between 0 Gy and 9.5 Gy. (PDF) [file pone.0252142.s014.pdf]

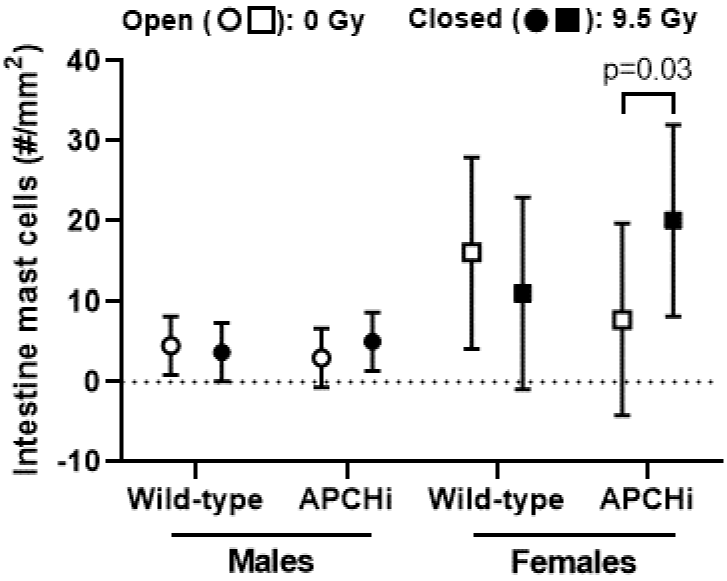

S14 Fig. Mast cell numbers in the intestinal wall at 6 months after irradiation. Mast cell numbers as determined from Toluidine Blue histology. Means and SD of the statistical model are shown; *n*=8 wild-type males in 0 Gy, 12 wild-type males in 9.5 Gy, 8 APCHi males in 0 Gy, 10 APCHi males in 9.5 Gy, 8 wild-type females in 0 Gy, 5 wild-type females in 9.5 Gy, 10 APCHi females in 0 Gy, and 9 APCHi females in 9.5 Gy. The bracket indicates a significant difference between 0 Gy and 9.5 Gy.
