# Supplementary material for: Sex-dependent effects of genetic upregulation of activated protein C on delayed effects of acute radiation exposure in the mouse heart, small intestine, and skin
Source: PLoS One. 2021 May 24;16(5):e0252142. doi: 10.1371/journal.pone.0252142 (PMC8143413; doi:10.1371/journal.pone.0252142)
Supplement: S15 Fig — Positivity for 4-HNE was assessed with immunohistochemistry. Graphs indicate means and SD of the statistical model; n = 6–9 wild-type males in 0 Gy, 10 wild-type males in 9.5 Gy, 6–8 APCHi males in 0 Gy, 8–9 APCHi males in 9.5 Gy, 7–8 wild-type females in 0 Gy, 5–6 wild-type females in 9.5 Gy, 8–9 APCHi females in 0 Gy, and 8–9 APCHi females in 9.5 Gy. (PDF) [file pone.0252142.s015.pdf]

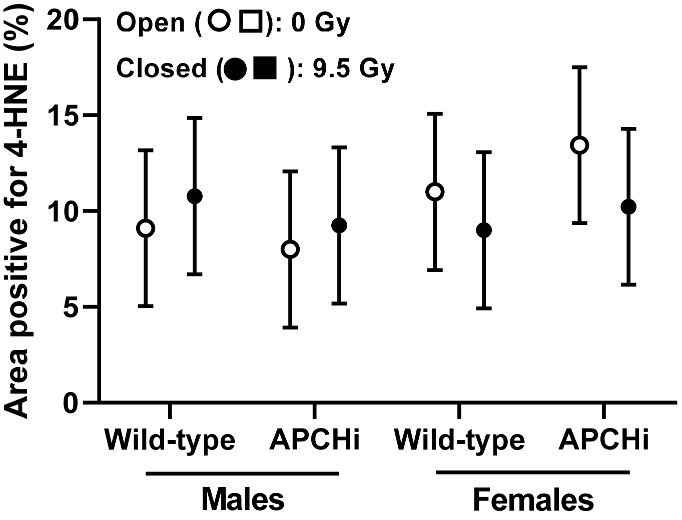

S15 Fig. Epidermal are positive for 4-HNE. Positivity for 4-HNE was assessed with immunohistochemistry. Graphs indicate means and SD of the statistical model; *n*=6-9 wild-type males in 0 Gy, 10 wild-type males in 9.5 Gy, 6-8 APCHi males in 0 Gy, 8-9 APCHi males in 9.5 Gy, 7-8 wild-type females in 0 Gy, 5-6 wild-type females in 9.5 Gy, 8-9 APCHi females in 0 Gy, and 8-9 APCHi females in 9.5 Gy.
