# Supplementary material for: Sex-dependent effects of genetic upregulation of activated protein C on delayed effects of acute radiation exposure in the mouse heart, small intestine, and skin
Source: PLoS One. 2021 May 24;16(5):e0252142. doi: 10.1371/journal.pone.0252142 (PMC8143413; doi:10.1371/journal.pone.0252142)
Supplement: S16 Fig — Arrow up: increase in irradiated animals compared to sham controls within the same genotype. Arrow down: decrease in irradiated animals compared to sham controls within the same genotype. Horizontal line: no significant effect of radiation. Shaded areas highlight differences in radiation response between the two genotypes. (PDF) [file pone.0252142.s016.pdf]

		♂ Wild-type	♂ APCHi	♀ Wild-type	♀ APCHi
Heart	Diameter (systole)	↑	↑	–	–
	Inner diameter (systole)	↑	↑	–	–
	Volume (systole)	↑	↑	–	–
	Ejection Fraction	↓	↓	–	–
	Fractional Shortening	↓	↓	–	–
	Stroke Volume	–	–	–	–
	LVPW thickness	↓	–	–	–
	Mitral valve E/A	–	–	↑	↑
	Heart rate	Not measured		–	–
	Collagen deposition	↑	↑	–	↑
	α-SMC actin positive cells	↑	↑	↑	↑
	Mast cell number	↓	↓	↓	↓
	TLR4 expression	↑	–	↑	–
	CD45 positive cells	↑	–	–	↑
	Microvascular density	↓	↓	↓	–
	vWf positive cells	↑	↑	↑	↑
Small intestine	MPO positive cells	–	–	↑	↑
	CD2 expression	↑	↑	–	–
	Collagen deposition	–	–	–	–
	α-SMC actin	–	–	–	–
	Mast cell numbers	–	–	–	–
Epidermis	4-HNE positive cells	–	–	–	–
	Epidermal thickness	↑	–	↑	–
	3-NT positive cells	↑	–	↑	–

S16 Fig. Graphic summary of research results obtained from male and female wild-type and APCHi mice at 6 months after 9.5 Gy compared to time-matched sham-irradiated controls. Arrow up: increase in irradiated animals compared to sham controls within the same genotype. Arrow down: decrease in irradiated animals compared to sham controls within the same genotype. Horizontal line: no significant effect of radiation. Shaded areas highlight differences in radiation response between the two genotypes.
