# Supplementary material for: Sex-dependent effects of genetic upregulation of activated protein C on delayed effects of acute radiation exposure in the mouse heart, small intestine, and skin
Source: PLoS One. 2021 May 24;16(5):e0252142. doi: 10.1371/journal.pone.0252142 (PMC8143413; doi:10.1371/journal.pone.0252142)
Supplement: S1 Table — (PDF) [file pone.0252142.s017.pdf]

S1 Table. Primary antibodies in immunohistochemistry and immunoblotting

Protein target	Host	Manufacturer (and clone)	Dilution
Antibodies in immunohistochemistry			
3-nitrotyrosine	Rabbit	EMD Millipore, Burlington, MA	1:1,000
4-hydroxynonenal	Rabbit	Abcam, Cambridge, MA	1:800
α-smooth muscle cell (SMC) actin	Rabbit	Abcam	1:1,000
CD45	Rat	Santa Cruz Biotechnology, Santa Cruz, CA (30-F11)	1:200
Von Willebrand factor	Rabbit	Santa Cruz Biotechnology (H-300)	1:25
Myeloperoxidase	Rabbit	Dako/Agilent, Troy, MA	1:100
Antibodies in immunoblotting			
αSMC actin	Rabbit	Abcam	1:4,000
CD2	Rabbit	M-180, Santa Cruz Biotechnology	1:2,000
CD45	Rat	30-F11, Santa Cruz Biotechnology	1:40,000
GAPDH	Mouse	6C5, Santa Cruz Biotechnology	1:20,000
Mast cell tryptase	Rabbit	FL-275, Santa Cruz Biotechnology	1:20,000
Toll-like receptor 4/MD2 complex	Mouse	MTS510, Thermo Fisher Scientific, Waltham, MA	1:1,000
