# Supplementary material for: Sex-dependent effects of genetic upregulation of activated protein C on delayed effects of acute radiation exposure in the mouse heart, small intestine, and skin
Source: PLoS One. 2021 May 24;16(5):e0252142. doi: 10.1371/journal.pone.0252142 (PMC8143413; doi:10.1371/journal.pone.0252142)
Supplement: S2 Table — The table shows detectable effect sizes, based on two sample sizes (n = 6 and n = 15) in each of the radiation×genotype×sex groups. (PDF) [file pone.0252142.s018.pdf]

S2 Table. Power analyses: Effect sizes detectable with 0.80 power on a 0.05 significance level. The table shows detectable effect sizes, based on two sample sizes (*n* = 6 and *n* = 15) in each of the radiation×genotype×sex groups.

n	**Simple**	**Interaction**
6 of one sex	1.66	2.36
15 of one sex	1.04	1.46

The effect sizes are expressed in SD units, where SD is the root mean square error assumed constant among all groups. The targeted power was 0.80 on 0.05 significance level *t*-tests conducted within the ANOVA or ANCOVA context. The simple effect is the difference in means between sham and 9.5 Gy irradiation for a given genotype. The interaction effect is the difference in the simple effects between APCHi and wild-type mice.
