# Supplementary material for: Sex-dependent effects of genetic upregulation of activated protein C on delayed effects of acute radiation exposure in the mouse heart, small intestine, and skin
Source: PLoS One. 2021 May 24;16(5):e0252142. doi: 10.1371/journal.pone.0252142 (PMC8143413; doi:10.1371/journal.pone.0252142)

Male heart α-smooth muscle cell (SMC) actin

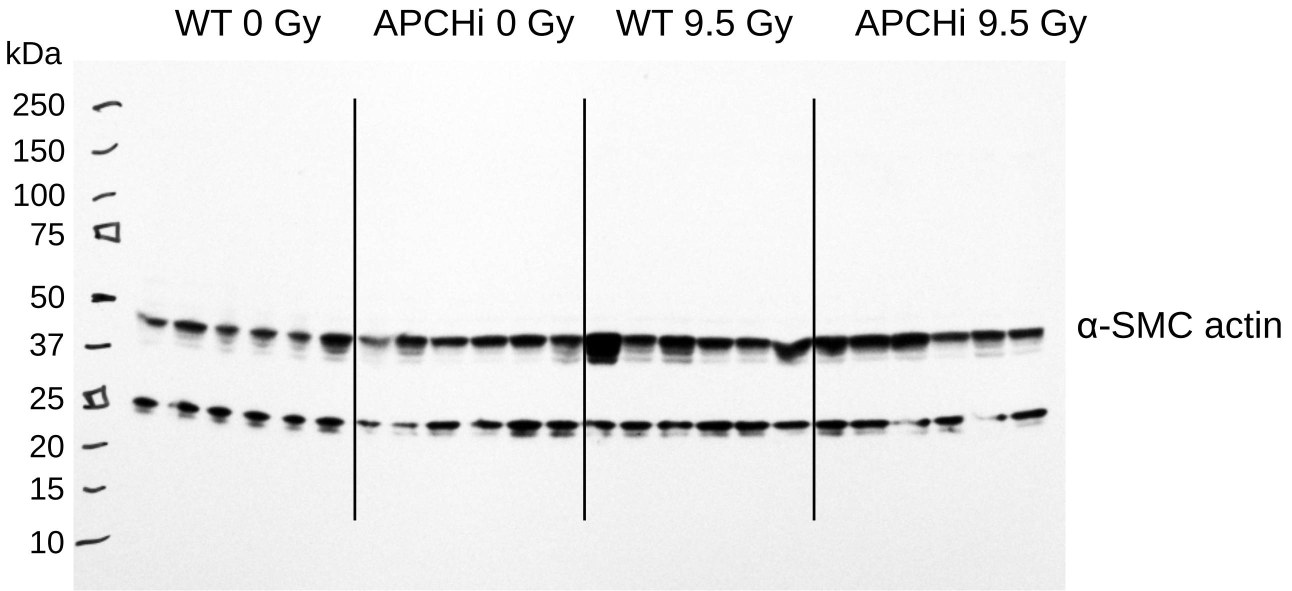

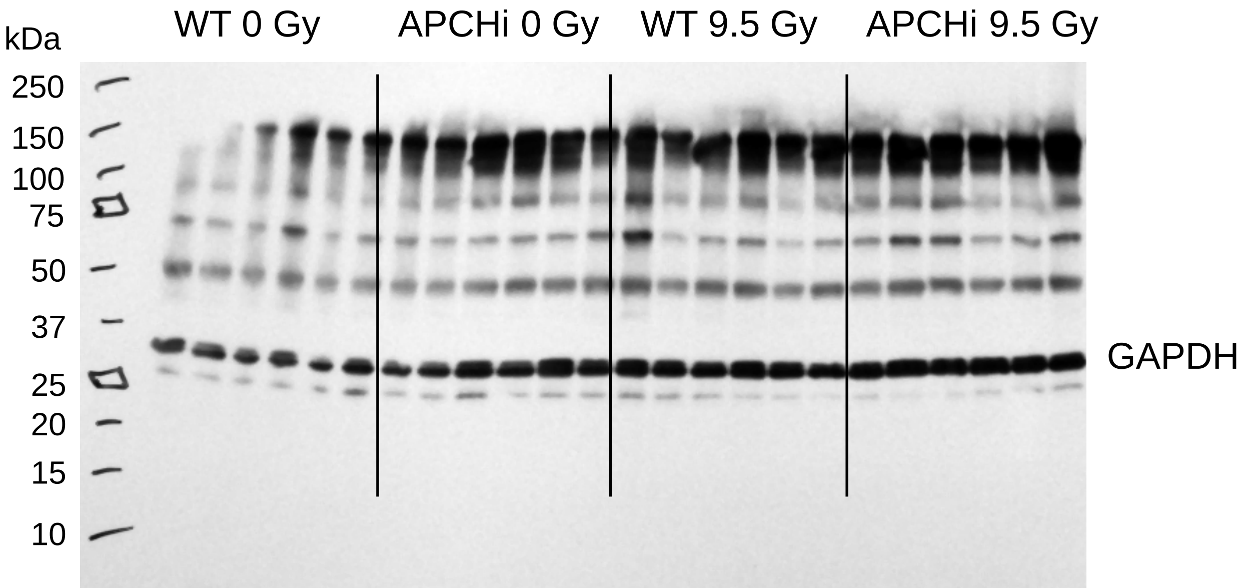

Female heart α-smooth muscle cell (SMC) actin

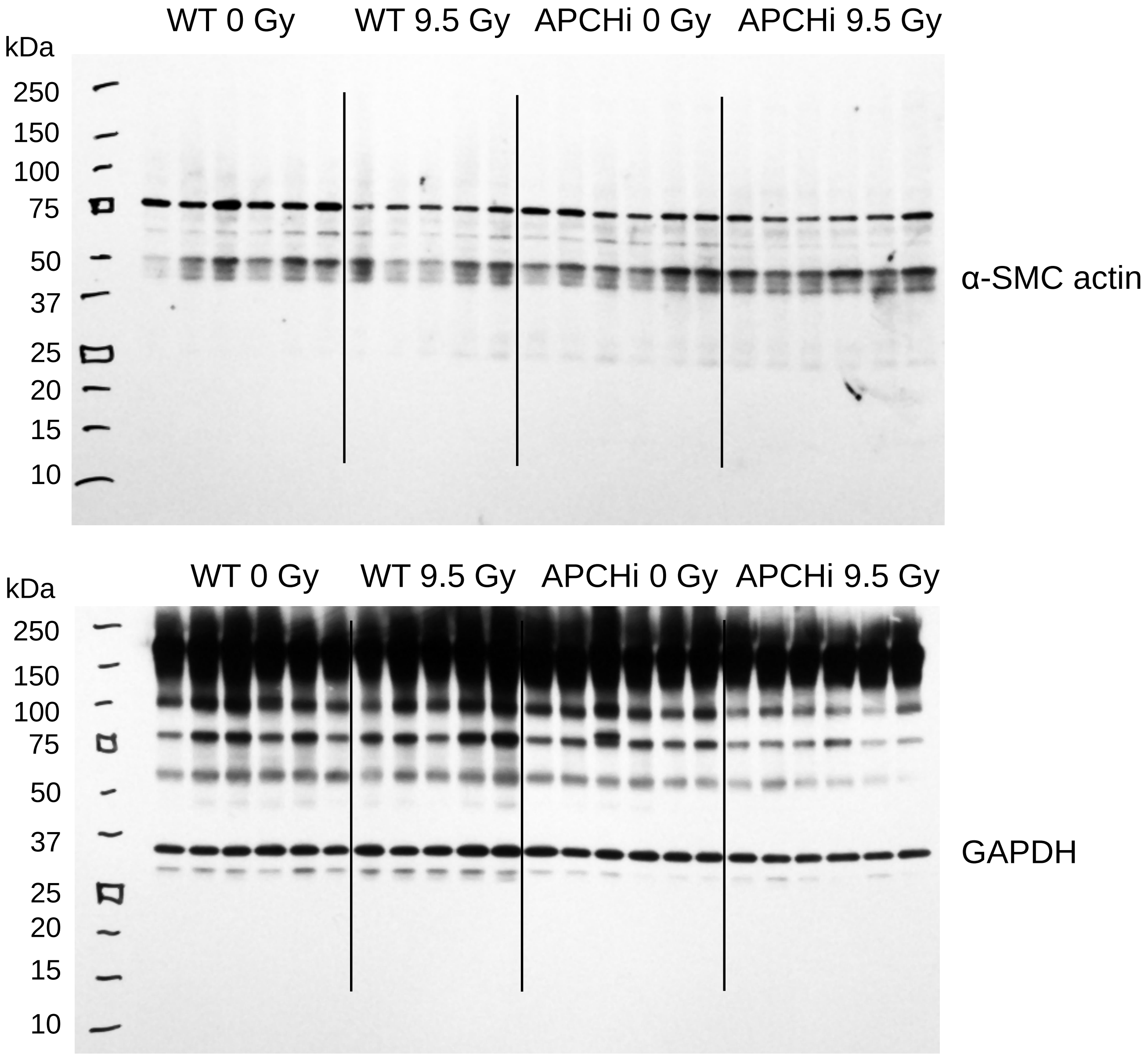

Male intestine α-smooth muscle cell (SMC) actin

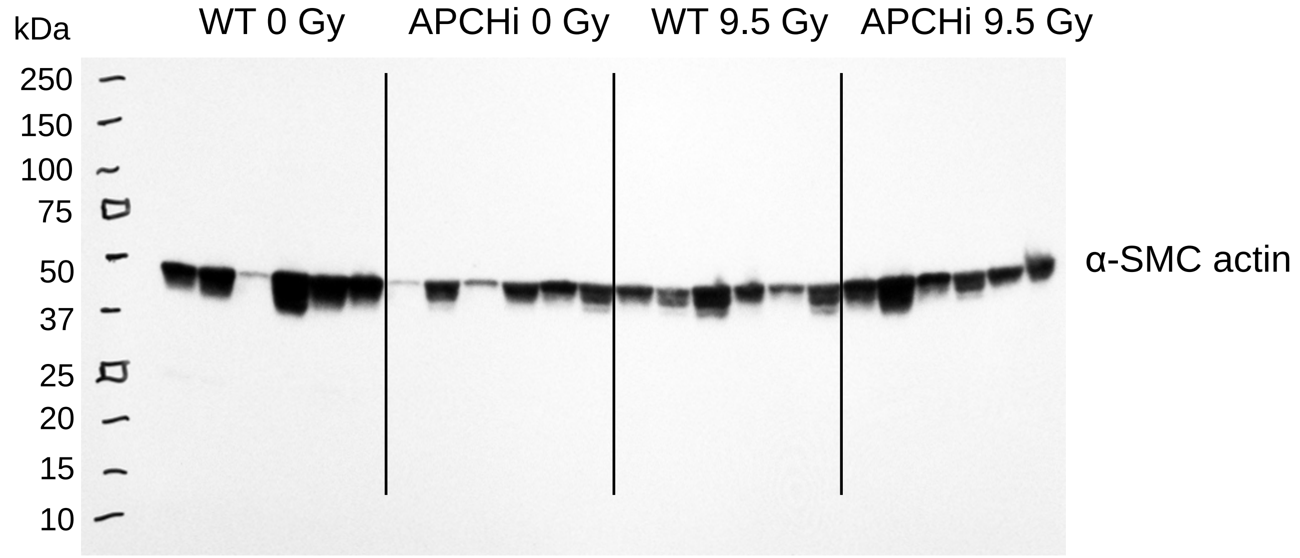

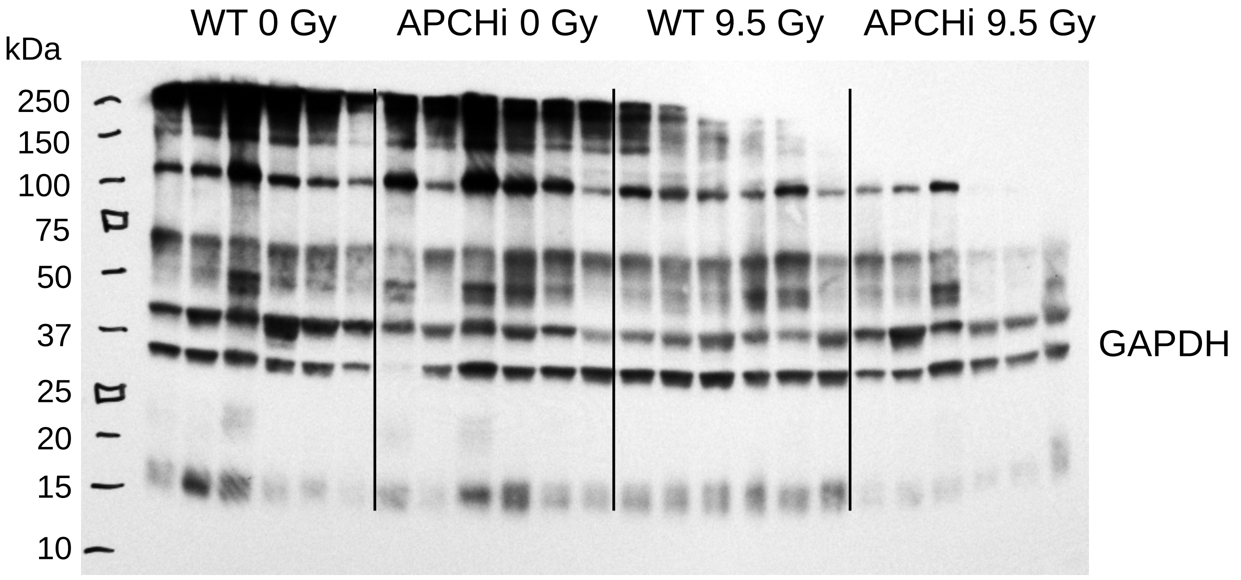

Female intestine α-smooth muscle cell (SMC) actin

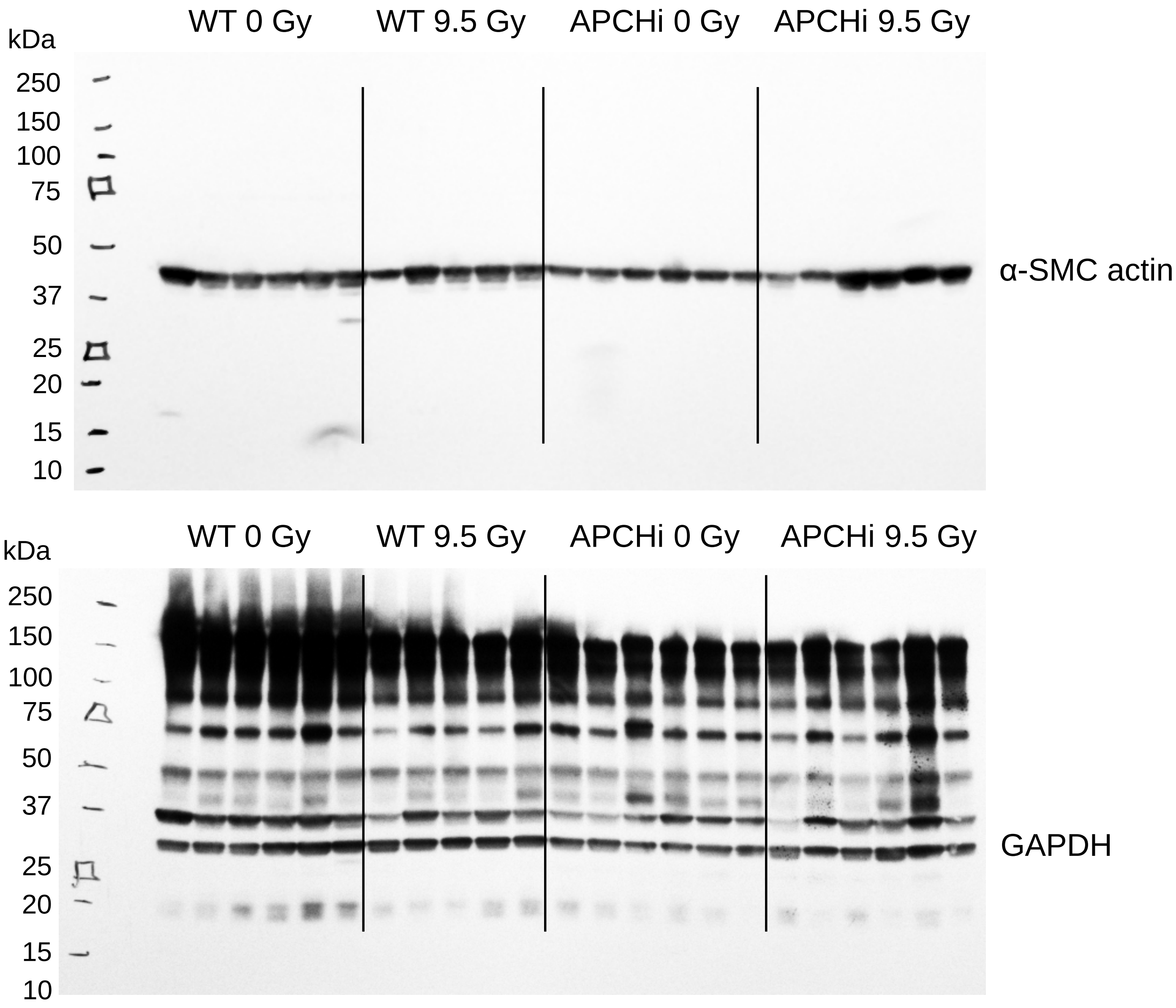

Male intestine CD2

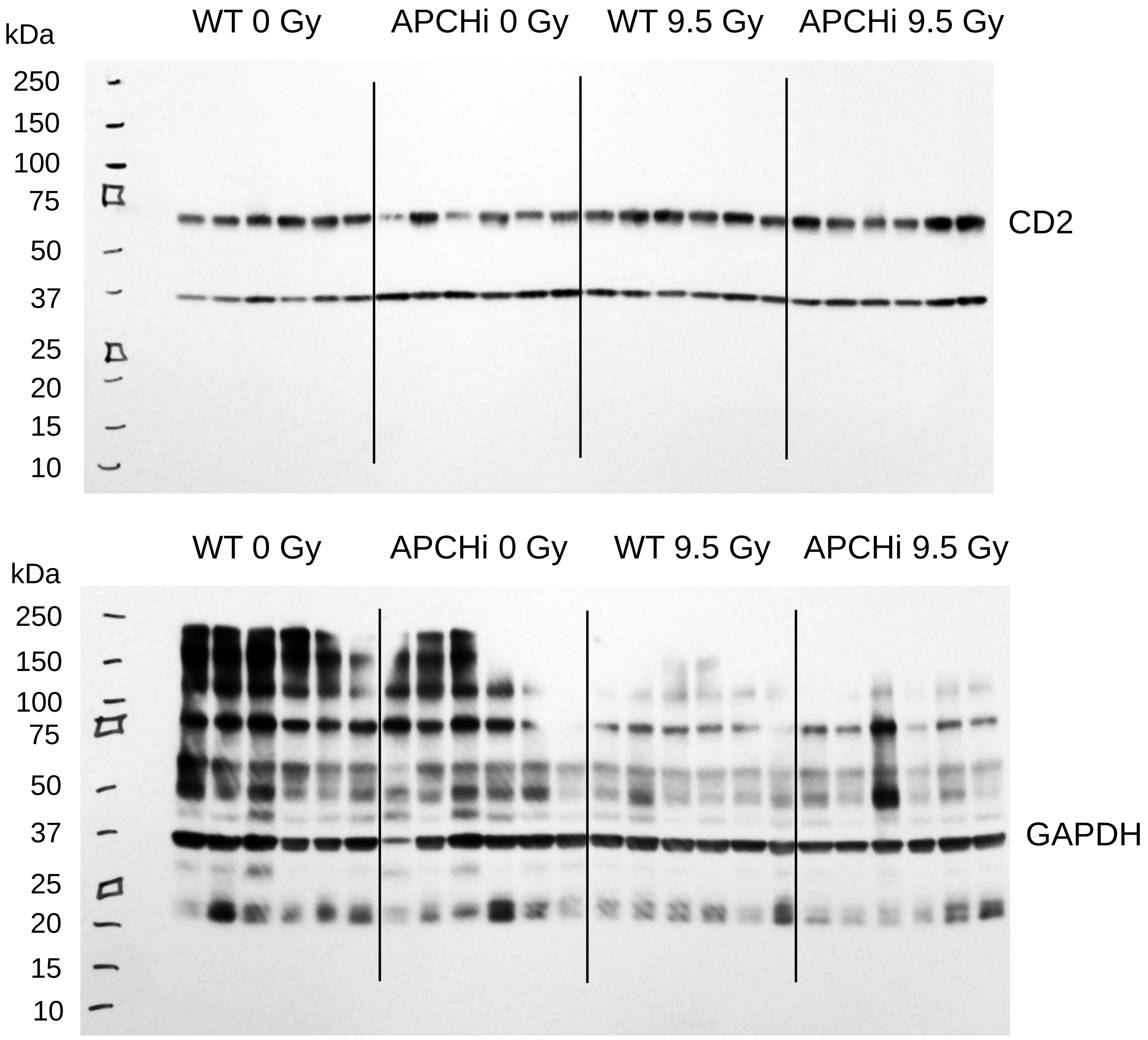

Female intestine CD2

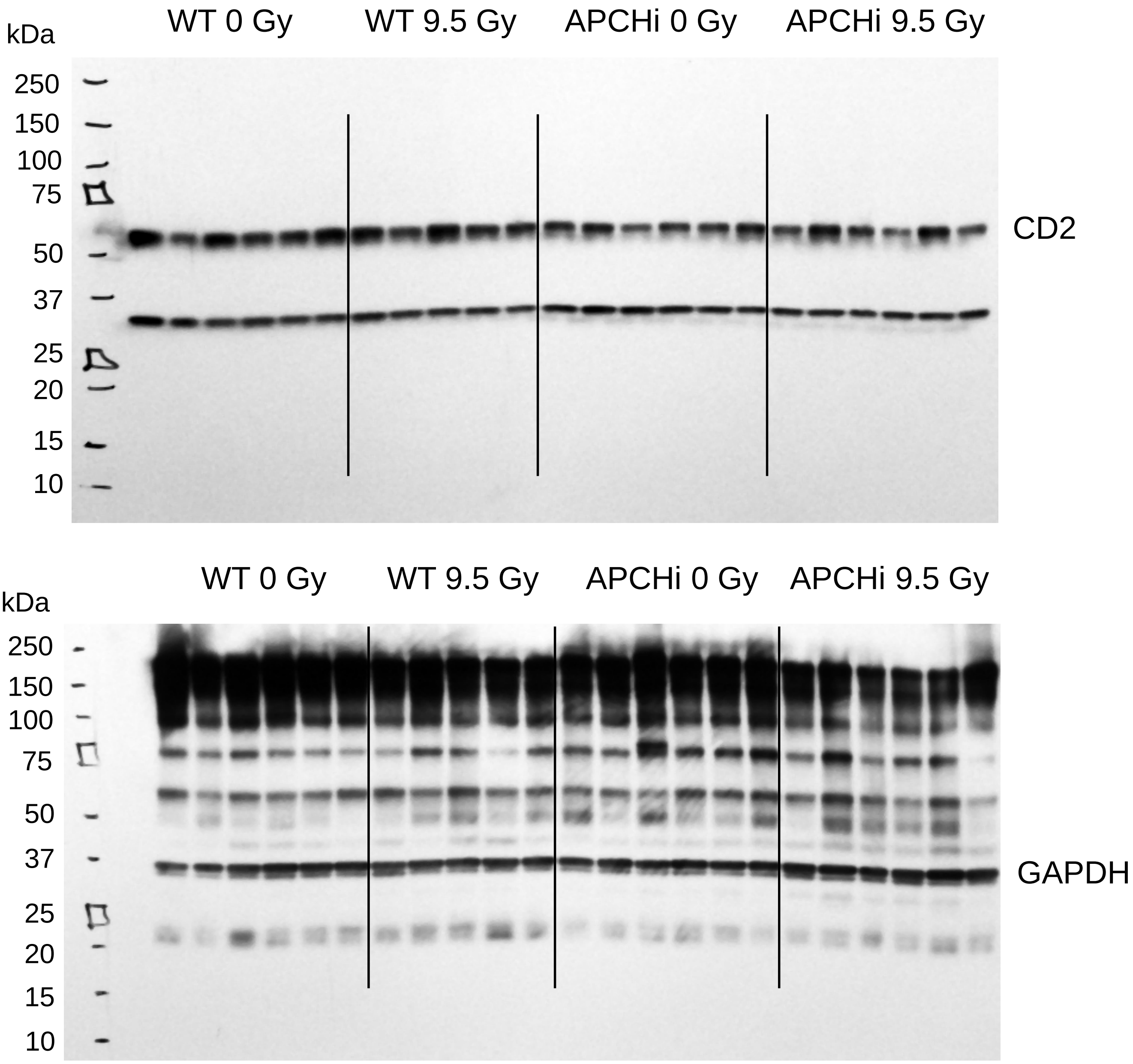

Male heart toll-like receptor 4 (TLR4)-MD2 complex

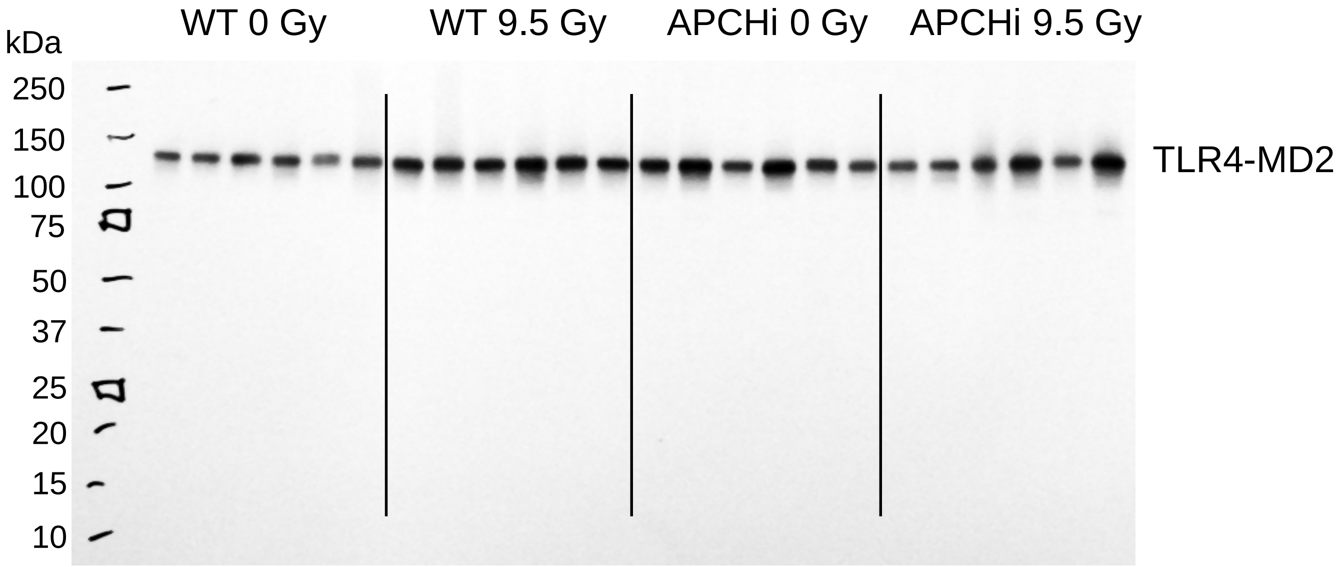

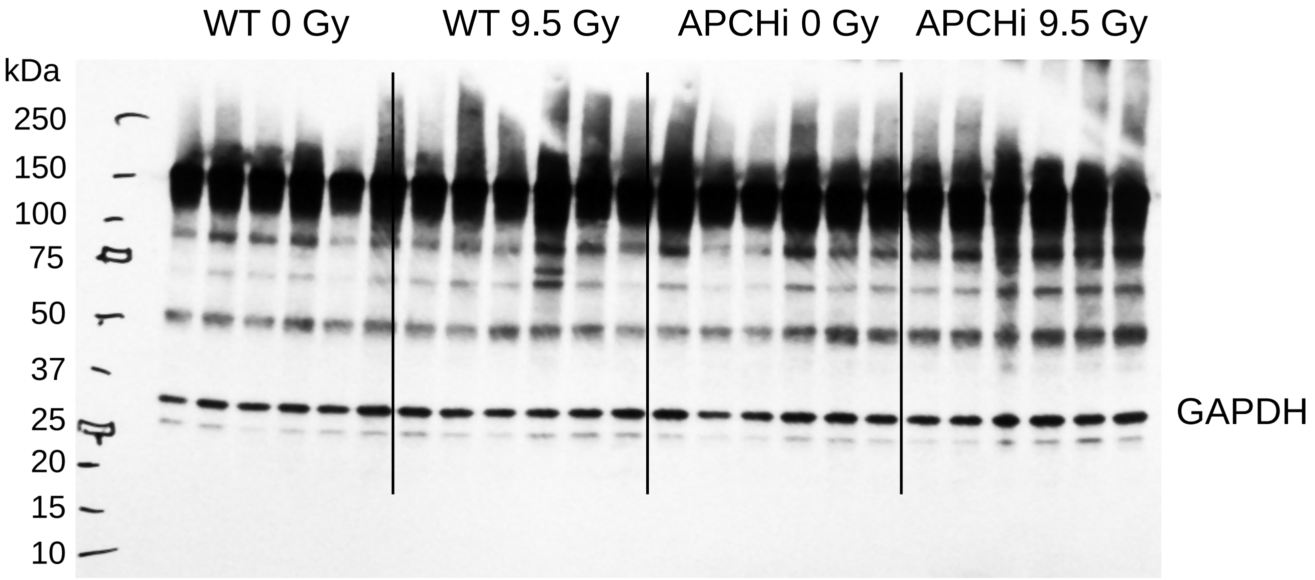

Female heart toll-like receptor 4 (TLR4)-MD2 complex

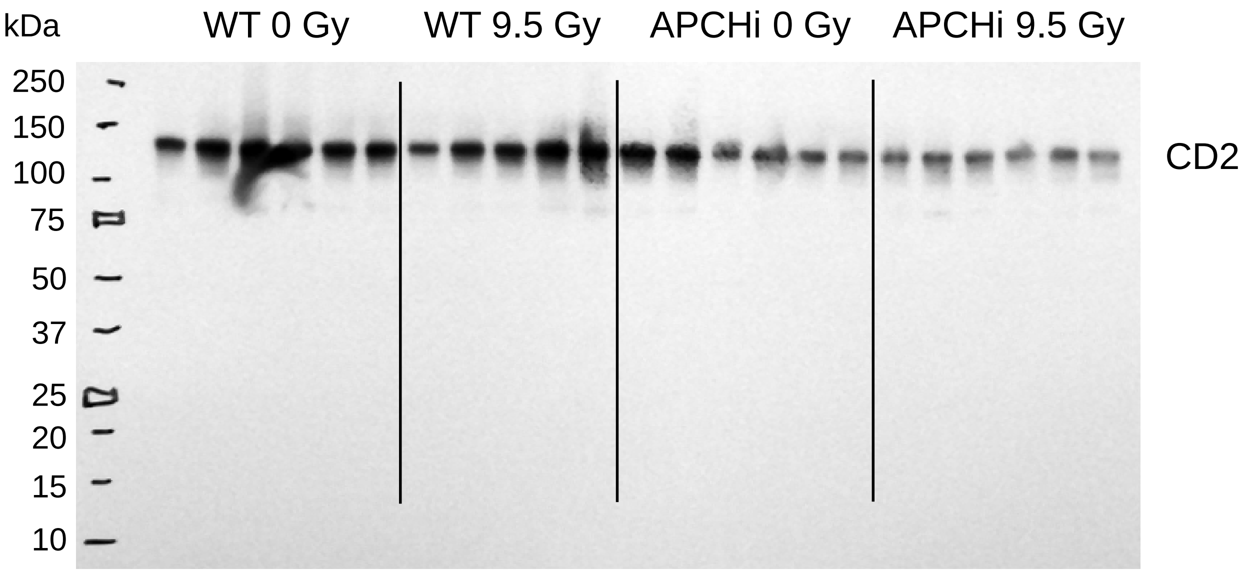

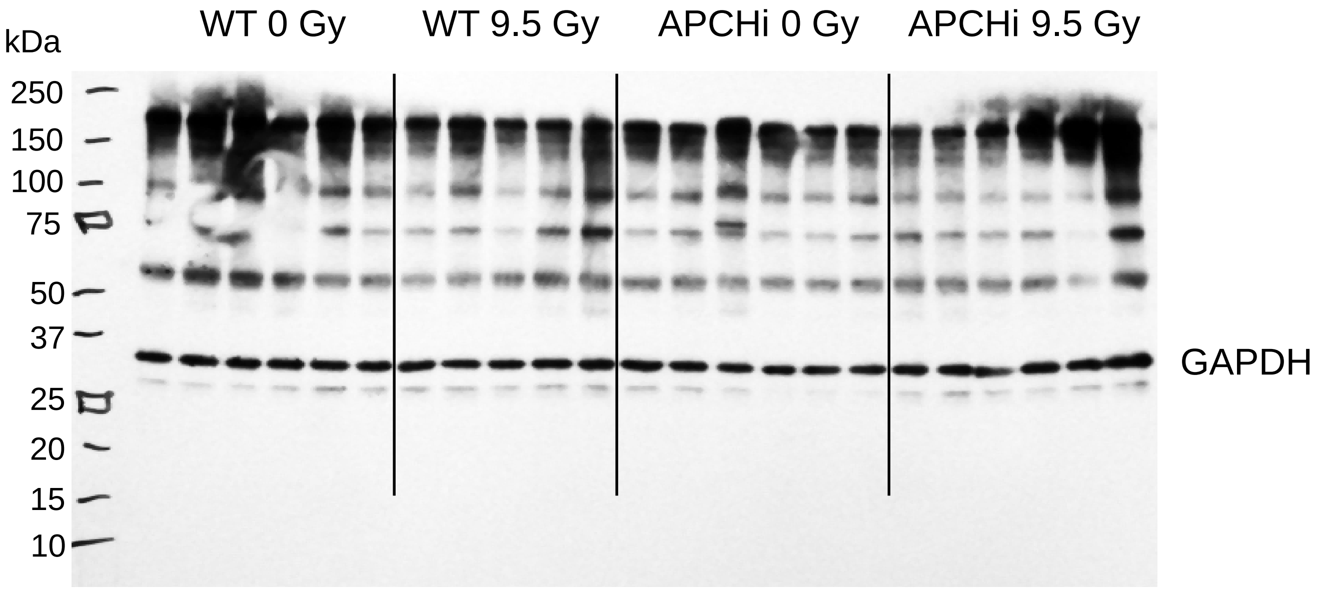

Supplement: S1 Raw images — (PDF) [file pone.0252142.s020.pdf]
